# Rhizosphere microorganisms of *Crocus sativus* as antagonists against pathogenic *Fusarium oxysporum*

**DOI:** 10.3389/fpls.2022.1045147

**Published:** 2022-11-22

**Authors:** Jiahao Zhang, Jiemiao Lu, Yichun Zhu, Qinger Huang, Luping Qin, Bo Zhu

**Affiliations:** School of Pharmaceutical Sciences, Zhejiang Chinese Medical University, Hangzhou, China

**Keywords:** *Crocus sativus*, corm rot, biocontrol activity, *Fusarium oxysporum*, rhizosphere microorganisms

## Abstract

**Introduction:**

Several microorganisms in the plant root system, especially in the rhizosphere, have their own compositions and functions. Corm rot is the most severe disease of *Crocus sativus*, leading to more than 50% mortality in field production.

**Methods:**

In this study, metagenomic sequencing was used to analyze microbial composition and function in the rhizosphere of *C. sativus* for possible microbial antagonists against pathogenic *Fusarium oxysporum*.

**Results:**

The microbial diversity and composition were different in the *C. sativus* rhizosphere from different habitats. The diversity index (Simpson index) was significantly lower in the *C. sativus* rhizospheric soil from Chongming (Rs_CM) and degenerative *C. sativus* rhizospheric soil from Chongming (RsD_CM) than in others. Linear discriminant analysis effect size results showed that differences among habitats were mainly at the order (Burkholderiales, Micrococcales, and Hypocreales) and genus (*Oidiodendron* and *Marssonina*) levels. Correlation analysis of the relative lesion area of corm rot showed that *Asanoa* was the most negatively correlated bacterial genus (ρ = −0.7934, *p*< 0.001), whereas *Moniliophthora* was the most negatively correlated fungal genus (ρ = −0.7047, *p*< 0.001). The relative lesion area result showed that *C. sativus* from Qiaocheng had the highest resistance, followed by Xiuzhou and Jiande. *C. sativus* groups with high disease resistance had abundant pathogen resistance genes, such as chitinase and β-1,3-glucanase genes, from rhizosphere microorganisms. Further, 13 bacteria and 19 fungi were isolated from *C. sativus* rhizosphere soils, and antagonistic activity against pathogenic *F*. *oxysporum* was observed on potato dextrose agar medium. *In vivo* corm experiments confirmed that *Trichoderma yunnanense* SR38, *Talaromyces* sp. SR55, *Burkholderia gladioli* SR379, and *Enterobacter* sp. SR343 displayed biocontrol activity against corm rot disease, with biocontrol efficiency of 20.26%, 31.37%, 39.22%, and 14.38%, respectively.

**Discussion:**

This study uncovers the differences in the microbial community of rhizosphere soil of *C. sativus* with different corm rot disease resistance and reveals the role of four rhizospheric microorganisms in providing the host *C. sativus* with resistance against corm rot. The obtained biocontrol microorganisms can also be used for application research and field management.

## 1 Introduction

The rhizosphere is a narrow soil zone affected by root secretions (Zhalnina et al., 2018). The soil microbial community is the largest biological resource pool known thus far (Torsvik et al., 2002), with its microbial species adding up to tens of thousands (Mahoney et al., 2017). Plants are closely related to microorganisms in rhizosphere soil. Rhizosphere microorganisms are essential in determining plant health, which may be related to microbial community structure and core microbial composition (Hou et al., 2021). Host plants may induce health beneficial microbes by the regulation of plant and microbiome signaling pathways (Gao et al., 2021), and this result is caused by their coevolution (Li et al., 2021b). Plant disease development may be affected by environmental factors, including commensal microbes, which either inhabit plant niches or live in soil (Kwak et al., 2018). Many beneficial microorganisms possess plant disease control activity in the rhizosphere; thus, revealing the secrets of microbial structure of the rhizosphere can help control plant diseases. For example, Hawaii 7996, a tomato variety resistant to the soil-borne pathogen *Ralstonia solanacearum*, possessed more abundant *Flavobacteriia*, Flavobacteriaceae, Sphingomonadaceae, and Pseudomonadaceae bacteria in the rhizosphere than the susceptible variety Moneymaker. Further, transplantation of rhizosphere microbiota from the resistant variety Hawaii 7996 could help the susceptible variety Moneymaker resist wilt, and the key strains may be the cause of the change and play an important role in disease resistance (Kwak et al., 2018). These results suggested that a good microbial composition can prevent pathogens from showing pathogenicity (Gordon, 2017; Zhou et al., 2019; Hu et al., 2020).

*Crocus sativus* L. (saffron) is a medicinal plant, whose stigma is used as valuable traditional Chinese medicine. The three main active components of *C. sativus* are crocin, picrocrocin, and safranal (Khorasanchi et al., 2018), which have an antidepressant effect (Dai et al., 2020) and antisenile dementia effect (Wang et al., 2019). However, *C*. *sativus* suffers from serious diseases in field production. The most severe disease of *C. sativus* is corm rot, leading to more than 50% mortality (Di Primo et al., 2002). Corm rot can be caused by various pathogens, but *Fusarium oxysporum* infection is the main cause (Hu et al., 2021). Various methods to manage plant rot diseases include chemical pesticide application, soil improvement technology, breeding of resistant varieties, and field rotation (Eshel et al., 2000; Shang et al., 2021; Dong et al., 2022; Wohor et al., 2022), but these methods are cumbersome with low efficiency and may cause severe environmental pollution. The interest in controlling plant diseases by beneficial microbes has recently increased because of the global need for environmentally friendly alternatives to chemical pesticides and fertilizers (Niu et al., 2020). Various microorganisms, such as phyllosphere microorganisms, endophytes, and rhizosphere microorganisms, can be used as biocontrol agents to prevent plant diseases (Vurukonda et al., 2018; Legein et al., 2020; Wang et al., 2021). Among these, biocontrol agents from rhizosphere microorganisms are the most common (Gu et al., 2020; Jiao et al., 2021). For example, rhizosphere bacteria *Pseudomonas chlororaphis*, *P. extremaustralis*, and *Acinetobacter lwoffii* A07 could alleviate damping-off disease caused by *Rhizoctonia solani* on *Pinus sylvestris* var. *mongolica* (Song et al., 2022).

Metagenomics is effective in revealing the microbiota related to pathogen infection and offers clues for the source of resistance against pathogens in plants (Mendes et al., 2011). Using metagenomic analysis, a previous study determined the diversity and abundance of bacteria and fungi in sour rot-affected table grapes, and confirmed that four of the isolated bacterial strains (two *Cronobacter* species, *Serratia marcescens*, and *Lysinibacillus fusiformis*) and five of the isolated fungal strains (three *Aspergillus* species, *Alternaria tenuissima*, and *F. proliferatum*) spoiled grapes (Gao et al., 2020). Many reports have analyzed the disease resistance of rhizosphere microorganisms to pathogens by co-culturing them with host plants. In a field experiment, inoculation with two antagonistic bacteria (*Pseudomonas* and *Bacillus*) in rhizospheric soil of tomato could stimulate its defense against *R. solani* (Kwak et al., 2018). Two rhizobacterial isolates *Bacillus subtilis* K4-4 and GH3-8 could completely suppress citrus dry root rot disease caused by *Neocosmospora solani* in greenhouse trials (Ezrari et al., 2021). The relationship between *C*. *sativus* rhizosphere microorganisms and phenology has been revealed by high-throughput sequencing (Ambardar et al., 2016); however, the relationship between the rhizosphere microbiome and disease resistance is unclear.

In this study, metagenomic tools were used to analyze the microbial composition in rhizosphere soil of *C. sativus* and predict gene function. Microorganisms related to the rot disease resistance of *C*. *sativus* were evaluated. We aimed to explore the relationship between the rhizosphere microbiome and host plant *C*. *sativus*, as well as the potential biocontrol fungal and bacterial strains against pathogenic *F. oxysporum*. These antagonistic microbes can be candidates for developing a microbial formulation for the biocontrol of corm rot disease of *C. sativus*.

## 2 Materials and methods

### 2.1 Sample collection and processing

*C*. *sativus* corm and its rhizosphere soil were collected from five distinct sites in China: Jiande, Zhejiang Province (29°32′34″N, 119°36′51″E), Xiuzhou, Zhejiang Province (30°39′41″N, 120°42′58″E), Chongming, Shanghai Municipality (31°39′59″N, 121°28′30″E), Chengcheng, Shaanxi Province (35°13′40″N, 109°57′5″E), and Qiaocheng, Anhui Province (33°37′33″N, 115°39′33″E). *C*. *sativus* corm was used for the disease resistance test, and rhizosphere soil was used for metagenomic analysis and microorganism isolation. The names and abbreviations of the groups are listed in **Table S1**. The loose soil around *C*. *sativus* corms was shaken off, and attached soil was brushed off gently and stored in an ice box. Plant tissue and gravel in rhizosphere soil were removed, and only rhizosphere soil was collected (Zhang et al., 2017).

Loose soil was air-dried, crushed, and passed through a screen mesh to assess chemical properties. Soil pH was determined with a digital pH meter (PHS-3E; Shanghai INESA & Scientific Instrument Co., Ltd., China) in a suspension of 1:2.5 soil/water ratio (w/v). Available nitrogen (AN) was determined by the alkaline hydrolysis diffusion method. Acidic available phosphorus (AP) and non-acid AP were extracted by NH_4_F–HCl solution (pH< 6.5) and NaHCO_3_ solution (pH ≥ 6.5), respectively, and the extracted AP was determined by the Mo-Sb anti-spectrophotometric method (Saudner, 1956). Available potassium (AK) was extracted by NH_4_OAc solution and determined by a flame photometer (F-500; Shanghai Metash Instruments Co., Ltd., China) (Shu et al., 2017). Soil organic matter (OM) was determined by the potassium dichromate oxidation method (Walkley and Black, 1934). Chromium (Cr) was determined by inductively coupled plasma mass spectrometry (X-2; Thermo Fisher Scientific, USA) after the soil samples were digested with HF–HNO_3_–H_2_O_2_.

### 2.2 Sequencing and metagenomic analysis

#### 2.2.1 Metagenomic sequencing and gene prediction

To reveal the *C*. *sativus* rhizospheric microbial structure, a high-throughput metagenomic sequencing approach was used. The rhizosphere soil in the previous step was used for DNA extraction and metagenomic sequencing. Metagenomic shotgun sequencing libraries were prepared and sequenced by Majorbio (Shanghai Majorbio Bio-pharm Technology Co., Ltd.) using the HiSeq 2000 platform. After quality control, the low-quality (<20 average quality) and N-containing reads were removed from the initial sequence data obtained from the metagenomic sequencing of *C*. *sativus* rhizosphere soil, and the high-quality sequences required for subsequent analysis were obtained. MEGAHIT was used for splicing, and the obtained contigs were predicted by open reading frame (ORF). MetaGene (http://metagene.cb.k.u-tokyo.ac.jp/) was used for ORF prediction (Hideki et al., 2006). Genes with nucleic acid lengths ≥100 bp were selected and translated into amino acid sequences. Redundancy was removed with CD-HIT (http://www.bioinformatics.org/cd-hit/) using bacterial and fungal redundant coding sequence catalogs. Sequences with ≥95% identity and 90% coverage were considered redundant (Fu et al., 2012).

#### 2.2.2 Species and functional analyses

Taxonomy information was matched against non-redundant (NR) protein databases (including Swiss-Prot, Protein Information Resource, Protein Research Foundation, Protein Data Bank, and the protein data translated from CDS data of GenBank and RefSeq) by DIAMOND (https://github.com/bbuchfink/diamond) (Buchfink et al., 2015; Buchfink et al., 2021) with E-value<1 × 10^−5^. Carbohydrate-active enzyme information was matched against the Carbohydrate-Active enZYme (CAZy) database (http://www.cazy.org/) by hmmscan (HMMER 3.0) with E-value<1 × 10^−5^ (Lombard et al., 2014). Gene function information was matched against the Kyoto Encyclopedia of Genes and Genomes (KEGG) database (http://www.genome.jp/kegg/) by DIAMOND with E-value<1 × 10^−5^ (Ogata et al., 1999).

#### 2.2.3 Statistical analysis

Alpha diversity, such as Simpson and Chao1 indices, was calculated by the number of reads of species using Quantitative Insights into Microbial Ecology 2 (QIIME2). The number of reads of species was also used for principal component analysis (PCA) based on the Euclidean distance by QIIME2. Adonis analysis was performed based on the Bray–Curtis distance and a significance test with 999 Monte Carlo test permutations in QIIME2 (Anderson, 2001). Also, linear discriminant analysis (LDA) effect size (LEfSe) was calculated by Kruskal–Wallis sum-rank test and LDA using LEfSe (http://huttenhower.sph.harvard.edu/galaxy/root?tool_id=lefse_upload) (Segata et al., 2011). Redundancy analysis (RDA) was performed by vegan in R (programming language), and the significance of RDA was judged by permutest analysis.

### 2.3 Isolation and identification of rhizosphere microorganisms

The pure-culture method was used to isolate microorganisms from rhizosphere soil (Jogaiah et al., 2013; Ezrari et al., 2021). The rhizosphere soil collected in Section 2.1 was used to isolate rhizosphere microorganisms. Serial dilutions were prepared up to 10^−5^ mg/mL using sterile water. Exactly 100 μL of each diluted sample was spread onto plates with nutrient agar (NA) medium (15 g peptone, 5 g NaCl, 3 g beef extract, 15 g agar, 1 L distilled water, and pH 7.2 ± 0.2) and potato dextrose agar (PDA) medium (200 g potato, 20 g glucose, 15 g agar, and 1 L distilled water). Plates with NA were incubated in the incubator (LMI-100; Shanghai Longyue Instrument Equipment Co., Ltd., China) at 28°C in the dark for 2 days, whereas plates with PDA were cultured at 26°C in the dark for 7 days. Bacterial colonies were transferred to fresh NA plates and purified by the streak plate method, whereas fungi were purified by constantly choosing hyphae and placing them on fresh PDA plates with an inoculating needle.

Identification of the purified isolates was achieved using 16S rRNA or ITS gene sequence analysis. First, total genomic DNA was extracted from bacteria or fungi using the TIANamp Bacteria DNA Kit (Tiangen, China) and DNAiso Reagent (TaKaRa Bio, China), respectively. Fungal ITS genes were amplified by polymerase chain reaction (PCR) with the primer pair ITS5 and ITS4, whereas bacterial 16S rRNA genes were amplified by PCR with the primer pair 27F and 1492R (Liu et al., 2019). PCR was performed in a 50 μL reaction mixture containing 2 μL of DNA, 1 μL forward primer (10 mM), 1 μL reverse primer (10 mM), 25 µL 2×Taq Mix (+Dye) (Monad Biotech, China), and 21 μL sterile PCR-grade water. The amplification procedure included an initial denaturation at 95°C for 5 min, followed by 30 cycles of 95°C for 30 s, 55°C for 30 s, 72°C for 1 min, and a final elongation step of 72°C for 10 min (Yang et al., 2020). The PCR amplified products were analyzed by agarose gel electrophoresis and sequenced at Sangon Biotech (Shanghai) Co., Ltd. (Shanghai, China). Bacteria and fungi were identified based on similarities to 16S rRNA and ITS sequences, respectively. The sequences of the isolates were searched against the NCBI database (https://www.ncbi.nlm.nih.gov/nuccore) and have been deposited in GenBank under the accession numbers listed in **Table S2**.

### 2.4 Disease resistance test of *C. sativus* corm

Disease resistance was evaluated by a *C*. *sativus* corm rot model. The corm rot disease model was constructed by injecting *C*. *sativus* with the corm rot pathogen (*F*. *oxysporum* CS60) spore solution. *F*. *oxysporum* CS60 was isolated from a typical rot *C*. *sativus* corm and identified based on the ITS rRNA gene sequence according to the methods in Section 2.3 (**Figure S1**). To satisfy Koch’s postulates, the causal fungus was reisolated from the lesions of inoculated *C*. *sativus* corm, with morphological and cultural characteristics, as well as ITS rRNA gene sequence identical to the original isolate (Ben et al., 2021). The spore solution was prepared as follows: three PDA disks containing 7-day-old *F*. *oxysporum* were inoculated into 30 mL sterilized potato dextrose broth (PDB), and the PDA disks were obtained by beating the edge of the colony with a cork borer (sterilized, 6 mm diameter). After culture in the shaker (MC-100B; Shanghai Muce Instrument Technology Co., Ltd., China) at 28°C and 180 rpm in the dark for 7 days, the spore suspension was obtained by filtering, and the spore concentration was adjusted to 10^6^/mL. *C*. *sativus* corms were injected with spore solution by a micro syringe, 30 μL each time, three injections with an interval of three days, and cultured in a greenhouse at 28 ± 2°C. *C*. *sativus* corm samples were tested for rot disease resistance by the relative lesion area (RLA) in the corm rot model. The corm area and lesion area were measured using AutoCAD 2019 software.


$$\text{RLA }\left(\%\right)=\frac{\text{lesion area}}{\text{corm area}}x 100\%$$

### 2.5 *In vitro* inhibition of *F. oxysporum*

Strains that could improve the corm rot resistance of *C. sativus* were screened. Based on microorganisms isolated from Section 2.3, the predicted active bacteria and fungi were evaluated for their antagonistic activity against *F. oxysporum*. The antagonism experiment was performed with PDA disks and filter paper (circle, 6 mm diameter). PDA disks containing 7-day-old *F. oxysporum* were placed at the center of PDA plates. Isolates with possible antagonism were placed 2.5 cm away from the center (three replicates per isolate). Specifically, 6 mm diameter PDA disks containing fungal threads were used in fungal antagonism, and filter papers containing 5 μL bacterial culture solution were used in bacterial antagonism. A week later, the diameter of the inhibition zone or inhibition rate was assessed (Liu et al., 2012).

### 2.6 *In vivo* evaluation of antagonistic microbes

For *in vivo* biocontrol evaluation of antagonistic microbes (*Trichoderma yunnanense* SR38, *Talaromyces* sp. SR55, *Burkholderia gladioli* SR379, and *Enterobacter* sp. SR343), antagonistic solutions were used. The antagonistic fungal solution was prepared as described in Section 2.4, whereas the antagonistic bacterial solution was prepared as follows. The bacterial colonies were scraped and placed in a flask containing 30 mL nutrient broth (NB). After culturing in the shaker at 28°C and 180 rpm in the dark for 2 days, the culture solution was filtered using a gauze and diluted to OD_600_ = 0.8 to obtain the antagonistic bacterial solution (Zu et al., 2022).

About 20 g healthy corms were selected for the experiment, and the antagonistic solutions were injected three times, 30 μL each time, with an interval of three days (day 0, 3, and 6). Pathogen spore solution was then injected three times, 30 μL each time, with an interval of three days (day 3, 6, and 9), and cultured in a greenhouse at 28 ± 2°C to investigate the incidence rate of corm rot. The blank group was injected with PDB or NB, and the positive control group was injected with carbendazim, 12 corms per group, repeated three times (Li et al., 2019).

Disease resistance was evaluated by the disease index, which was calculated as follows (Tian et al., 2021a):


$$\text{Disease index=}\frac{\sum^{}\left(\text{Number of each level disease corm×Values of each level}\right)}{\text{Total number of corms×Top level value}}\times 100\%$$

Level 0: no disease, Level 1: RLA ≤ 20%, Level 2: 20%< RLA ≤ 40%, Level 3: 40%< RLA ≤ 60%, Level 4: 60%< RLA ≤ 80%, Level 5: 80%< RLA ≤ 100%.

## 3 Results

### 3.1 Microbial species composition and differential analysis

Soil DNA was sheared into 500 bp fragments, amplified, and sequenced to obtain an average of 93 729 748 raw reads. To build a high-quality sequencing library, an average of 91 247 300 clean reads were obtained from raw reads by quality control, and the percentage in raw reads (%) was 97.35%. The high-quality sequences were then assembled into contigs (average 890 335). Finally, ORFs were taken for analysis after ORF prediction (**Table S1**).

**Figure 1:**
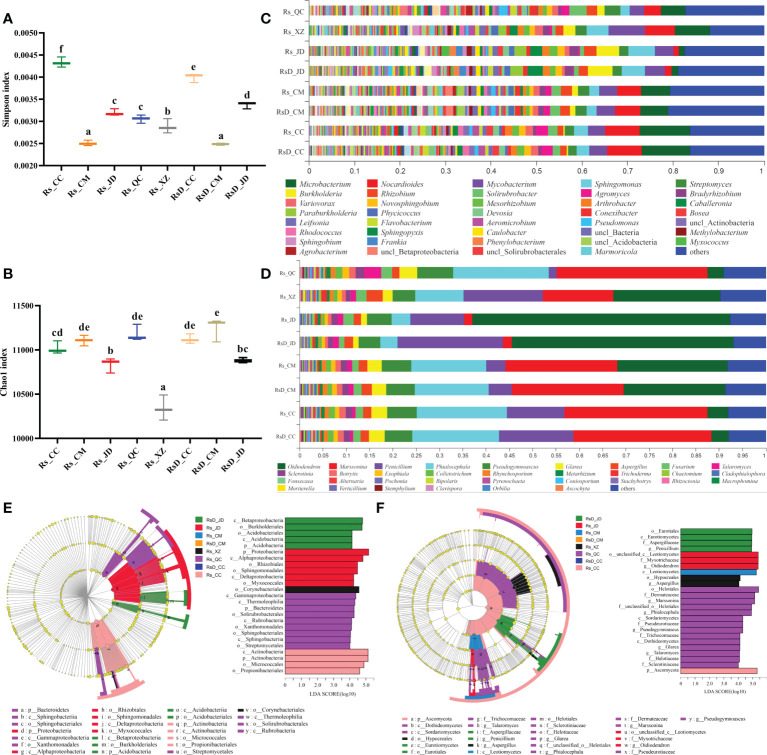
Rhizosphere microorganism composition of *Crocus sativus* from different origins. Boxplot of alpha-diversity indices: **(A)** Shannon index; **(B)** Chao1 index (group significance is indicated by letters; non-identical letters indicate *p*< 0.05). **(C)** Relative abundances of bacterial genera in samples. **(D)** Relative abundances of fungal genera in samples. **(E)** Cladogram generated from LEfSe analysis showing the most differentially abundant bacterial taxa enriched in rhizosphere with LDA scores (LDA score > 4 are shown). **(F)** Cladogram generated from LEfSe analysis showing the most differentially abundant fungal taxa enriched in rhizosphere with LDA scores (LDA score > 4 are shown).

Among these, the analyzed NR gene set was constructed by the sequences related to bacteria and fungi, and the gene sequence numbers were 9 033 718 and 128 495, respectively. The annotation results with the NR database showed that the genes in the gene set belonged to 91 phyla, 170 classes, 335 orders, 658 families, 2 268 genera, and 13 789 species. To evaluate the abundance and diversity of microbial communities, the diversity index (Simpson index) and richness index (Chao1 index) were calculated based on species (**Figures 1A, B**). The Simpson index was significantly lower in Rs_CM and RsD_CM than in others. Rs_XZ had a significantly lower Chao1 index among all the groups. Although rhizosphere microbial diversity was often related to host conditions, specific microbes in the microbiome could play key roles.

The microbial species composition was also analyzed. Bacterial composition could be divided into two categories, with either (*Microbacterium* + *Nocardioides*) or (*Burkholderia* + *Sphingomonas*) as the most dominant genus. Rs_JD had the highest abundance of *Sphingomonas* (5.83%), and Rs_CC had the highest abundance of *Microbacterium* (11.14%). In addition, a higher abundance of *Mycobacterium* was found in Rs_XZ, reaching 8.11%, which was 2.06 times that of RsD_CC (**Figure 1C**). Fungal samples could be divided into two categories, with the most dominant genus as either *Oidiodendron* or *Marssonina*. The abundance of *Oidiodendron* in Rs_JD was the highest and even up to 55.35%, which was 13.98 times higher than RsD_CC. The abundance of *Talaromyces* and *Pseudogymnoascus* in Rs_QC was unusual and up to 3.52% and 7.76%, which were 2.83 and 1.30 times as large as in RsD_CC, respectively (**Figure 1D**).

Dissimilarity among samples was explored using PCA, and the compositions of the groups were distinct from each other (**Figure S2**, PC1 = 59.23%, PC2 = 19.11%), which was similar to the Adonis analysis (sample variation 0.9785, *p* = 0.001). The different taxon units were confirmed by LEfSe analysis, and Burkholderiales was enriched in RsD_JD, whereas Micrococcales was enriched in Rs_CC at the order level, and Proteobacteria with the highest LDA (5.17) was enriched in Rs_JD at the phylum level (LDA > 4, **Figure 1E**). For fungi, LefSe results revealed that *Marssonina* was enriched in Rs_QC, whereas Ascomycota was enriched in Rs_CC. Moreover, Helotiales, *Oidiodendron*, and Myxotrichaceae were the groups with top three LDA. *Oidiodendron* and Myxotrichaceae were enriched in Rs_JD, whereas Helotiales was enriched in Rs_QC (LDA > 4, **Figure 1F**).

### 3.2 Correlations among taxonomic levels, soil physicochemical properties, and disease resistance

**Figure 2:**
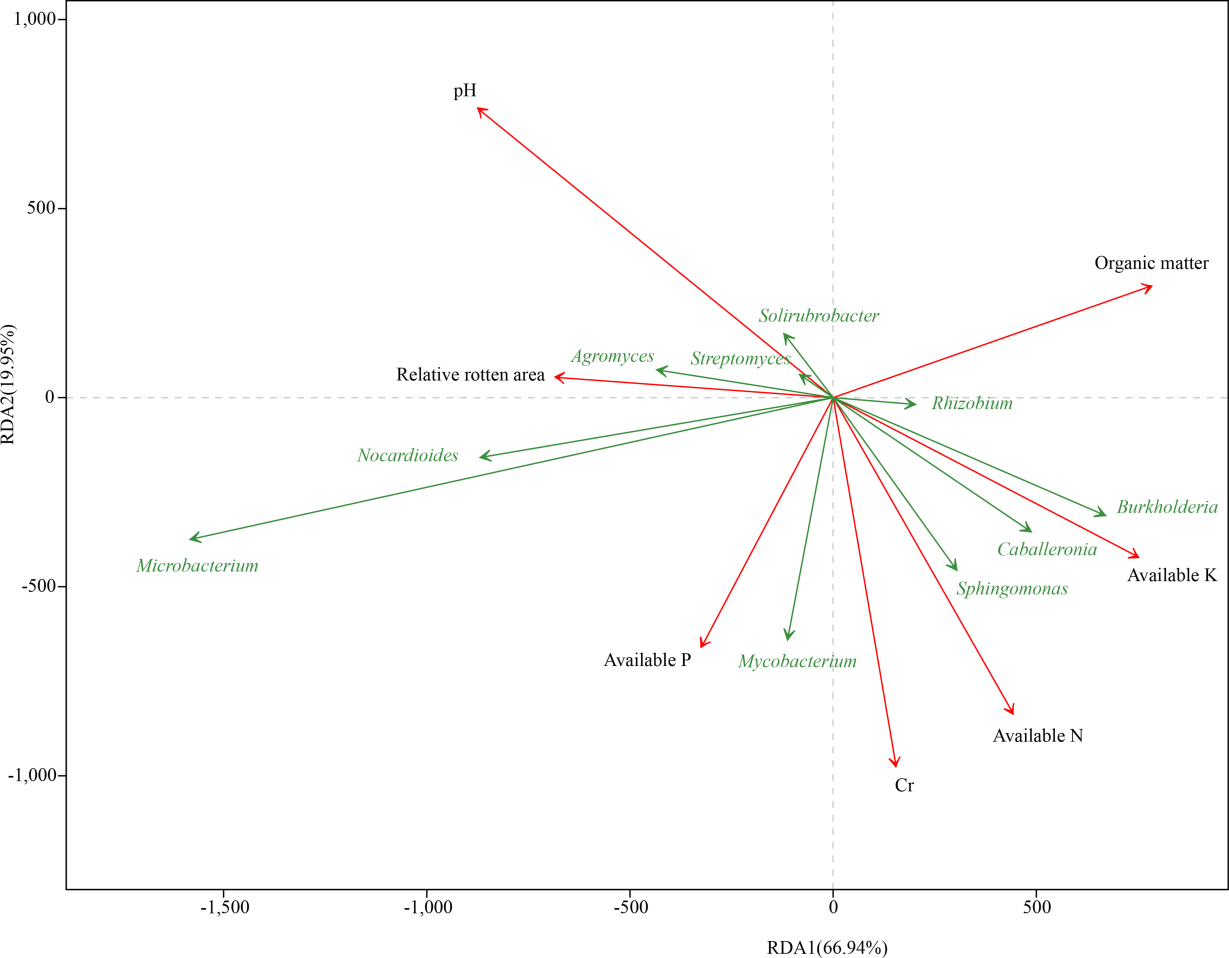
Redundancy analysis of rhizospheric genera corresponding to soil and corm properties. The 10 best-fitting genera are shown.

To evaluate the resistance of different *C*. *sativus* corms to *F*. *oxysporum*, samples of eight groups from five regions were tested. RLA denoted the incidence rate of corm rot in *C*. *sativus*. The RLA result showed that RsD_CC had the highest incidence rate (RLA = 67.17 ± 5.02%), followed by RsD_CM (48.17 ± 2.06%), Rs_CC (40.20 ± 3.37%), RsD_JD (35.66 ± 3.12%), Rs_CM (34.32 ± 3.84%), Rs_JD (25.50 ± 0.66%), Rs_XZ (25.35 ± 6.12%), and Rs_QC (22.29 ± 3.87%), indicating that Rs_QC had the highest resistance (**Table S3**, **Figure S3**). There was a close relationship between taxonomic levels and disease resistance. Specifically, the top 10 genera in abundance were revealed by RDA (**Figure 2**). RLA significantly affected the bacterial community (r^2^ = 0.32, permutest *p*< 0.05), which was negatively correlated with the abundance of *Burkholderia* and positively correlated with the abundance of *Agromyces*.

The determined soil chemical properties (**Table S4**) were used for RDA, and a significant correlation was found between soil chemical properties (pH, AN, AP, AK, OM, and Cr) and taxonomic levels (**Figure 2**). pH was the closest environmental factor related to microbial communities (r^2^ = 0.94, permutest *p*< 0.001), followed by Cr (r^2^ = 0.67, permutest *p*< 0.001) and AN (r^2^ = 0.62, permutest *p*< 0.001) (**Table S5**), and it was most positively correlated with the abundance of *Microbacterium* (**Figure 2**). The abundance of *Burkholderia*, *Caballeronia*, *Mycobacterium*, *Sphingomonas*, and *Rhizobium* was positively correlated with Cr and AN. In addition, pH had the closest correlation with RLA, showing a positive correlation, whereas the strongest negative correlation was with AK, according to the angle between the vectors of representative factors.

**Figure 3:**
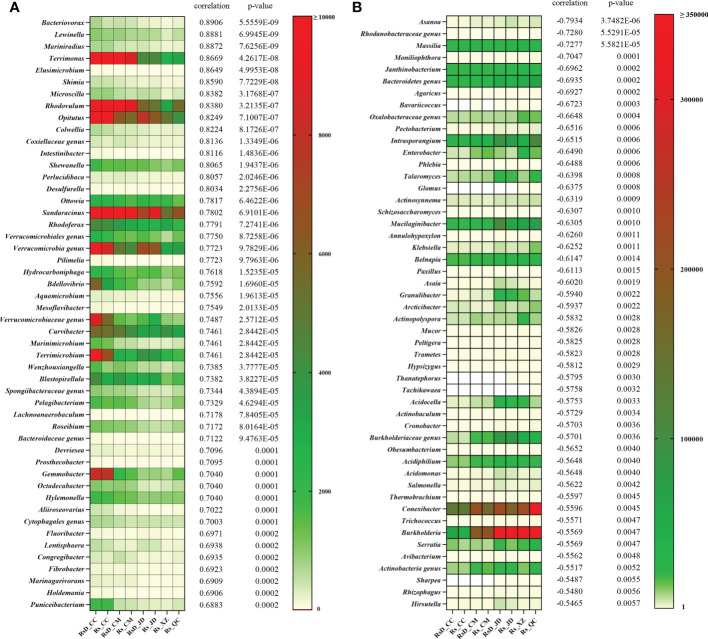
Top 50 rhizospheric genera correlations with relative lesion area. **(A)** Positive correlation and **(B)** negative correlation.

To clarify a deeper relationship between RLA and taxa, Spearman’s correlation analysis was performed. A total of 2 269 genera were used in the analysis, including 586 genera with significant correlation in which 384 genera were positively correlated and 202 genera were negatively correlated (**Table S6**). Moreover, the top 50 close genera with both positive and negative correlations are shown in **Figure 3**. Among the genera with positive correlation, *Bacteriovorax* had the greatest correlation (ρ = 0.8906, *p*< 0.001), followed by *Lewinella* (ρ = 0.8881, *p*< 0.001), *Mariniradius* (ρ = 0.8872, *p*< 0.001), and *Terrimonas* (ρ = 0.8669, *p*< 0.001). Among the genera with negative correlation, the bacterial genus *Asanoa* had the closest correlation (ρ = −0.7934, *p*< 0.001), whereas the closest fungal genus was *Moniliophthora* (ρ = −0.7047, *p*< 0.001). In addition, genera negatively correlated with RLA included many microbes of disease resistance, such as *Massilia* (ρ = −0.7277, *p*< 0.001) (Li et al., 2021a), *Talaromyces* (ρ = −0.6398, *p*< 0.001) (Tian et al., 2021c), *Burkholderia* (ρ = −0.5569, *p*< 0.01) (Ahmad et al., 2022), etc.

### 3.3 Metagenomic analysis of rhizosphere microorganism functions

**Figure 4:**
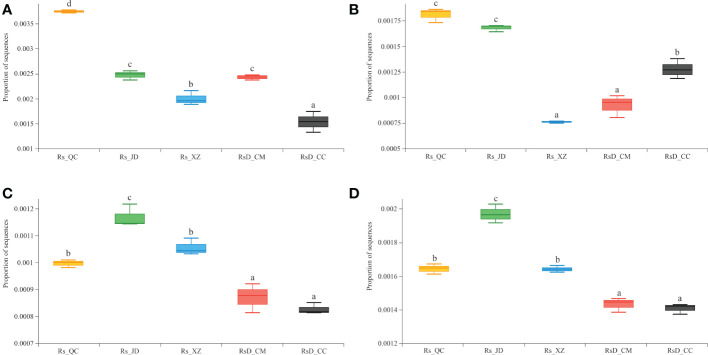
Boxplot of functional gene sequences proportion. **(A)** chitinase; **(B)** β-1,3-glucanase; **(C)** spermidine/putrescine transport system substrate-binding protein; and **(D)** aldehyde dehydrogenase (group significance is indicated by letters; non-identical letters indicate *p*< 0.05).

To understand the gene functions of rhizosphere microorganisms in resistance to different diseases, the CAZy and KEGG databases were used for annotation. Further exploration of the metagenomic data revealed a higher abundance of sequences associated with chitinase (GH19, EC 3.2.1.14) and β-1,3-glucanase (GH128, EC 3.2.1.39). These were usually considered defense enzymes against pathogens in Rs_QC or Rs_JD. In particular, chitinase was significantly higher in Rs_QC than in other groups (*p*< 0.05), even 2.43 times as high as in RsD_CC (**Figure 4A**). Although the levels of β-1,3-glucanase in Rs_QC and Rs_JD were excellent, they were lower in Rs_XZ with good disease resistance (**Figure 4B**). Moreover, a higher abundance of sequences was associated with the spermidine/putrescine transport system substrate-binding protein (K02055) in Rs_QC, Rs_JD, and Rs_XZ, which were significantly higher than RsD_CC and RsD_CM (**Figure 4C**). Meanwhile, a higher abundance of sequences focused on aldehyde dehydrogenase (K00128, EC 1.2.1.3) in Rs_JD (**Figure 4D**), which was significantly higher than in RsD_CM and RsD_CC (*p*< 0.05). These functions were related to quorum sensing and biosynthesis of polyketides, which might increase competition to hinder pathogen invasion (**Table S7**, **S8**).

### 3.4 Biocontrol activity evaluation of microbial antagonists

**Figure 5:**
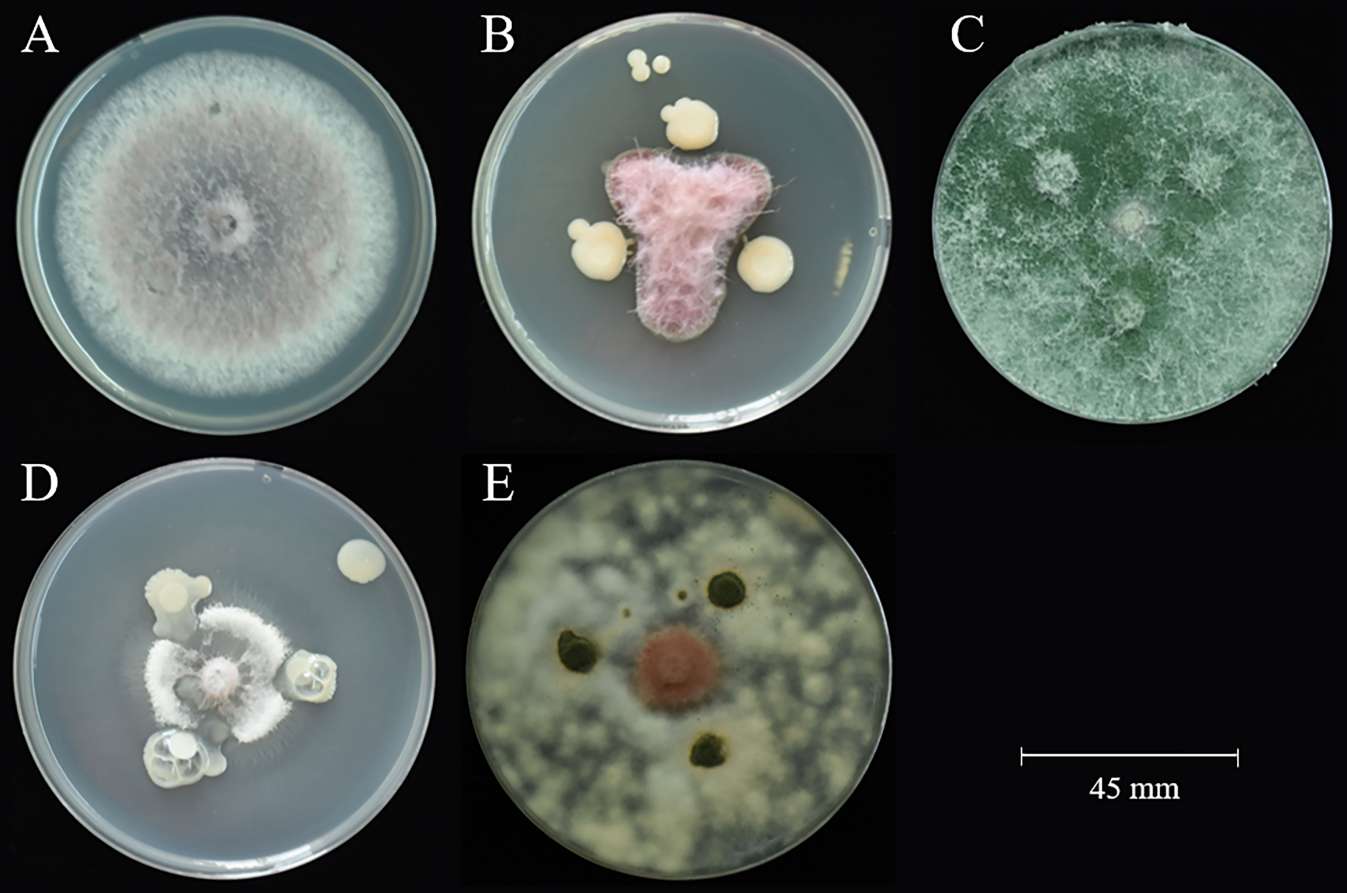
Antagonistic activity against *F. oxysporum*. **(A)** control *F. oxysporum*; **(B)** SR379 vs. *F. oxysporum*; **(C)** SR38 vs. *F. oxysporum*; **(D)** SR343 vs. *F. oxysporum*; **(E)** SR55 vs. *F. oxysporum*.

**Figure 6:**
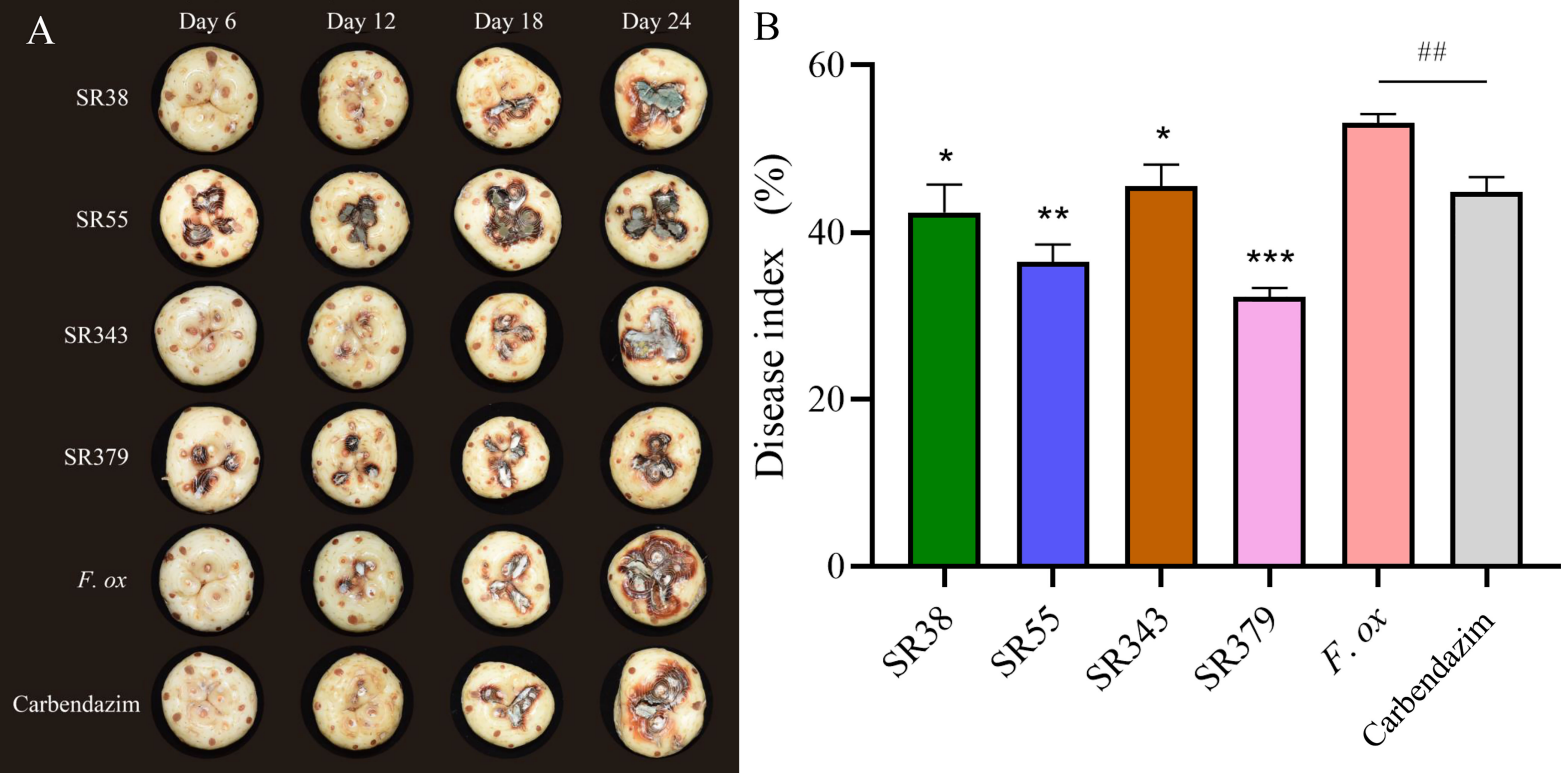
Evaluation of corm rot disease resistance of rhizosphere microorganisms *in vivo*. **(A)** Photographs of lesions on corm. **(B)** Histogram of disease index (SR38: *Trichoderma yunnanense* SR38 + *Fusarium oxysporum*; SR55: *Talaromyces* sp. SR55 + *F*. *oxysporum*; SR343: *Enterobacter* sp. SR343 + *F*. *oxysporum*; SR379: *Burkholderia gladioli* SR379 + *F*. *oxysporum*; *F. ox*: Sterile medium + *F. oxysporum*; and Carbendazim: 0.125% carbendazim solution + *F. oxysporum*) (group significance is indicated by * or ^#^: ^∗^ *p*< 0.05, ^∗∗^ *p*< 0.01, ^∗∗∗^ *p*< 0.001, ^##^ *p*< 0.01).

A total of 65 bacteria and 41 fungi were isolated from *C. sativus* rhizosphere soils. By the functional prediction of microorganisms (**Table S6**), 13 bacteria and 19 fungi were selected as potential microbial antagonists for biocontrol activity experiments. Of these, 26 (81.25%) showed inhibitory effects on *F. oxysporum* on PDA plates (**Table S2**; **Figure 5**). The top two fungi and bacteria, *T. yunnanense* SR38, *Talaromyces* sp. SR55, *B. gladioli* SR379, and *Enterobacter* sp. SR343, were chosen to test *in vivo C. sativus* corm (**Figure 6A**). SR379 had the best defense effect against pathogenic *F. oxysporum*, and its RLA was 30.10%. The group with only *F*. *oxysporum* was seriously diseased, and RLA was 1.77 times higher than SR379, even reaching 53.41% (**Table S9**). The disease index was evaluated after inoculating microbial antagonists, which showed that treating SR38, SR55, SR343, and SR379 had good biocontrol effect, with biocontrol efficiency of 20.26%, 31.37%, 14.38%, and 39.22%, respectively. Biocontrol activities of all antagonistic groups were higher than those of the pesticide group (**Figure 6B**).

## 4 Discussion

The plant rhizosphere is an essential environment, which is affected by root exudates (Sasse et al., 2018); therefore, microorganisms in the rhizosphere, including bacteria and fungi, are more closely related to plant life activities compared with field soil. In particular, the relationship between rhizosphere microorganisms and plant health has been a research hotspot. A previous study investigated the relationship between key rhizosphere microorganisms and traits of *Arabidopsis*, and found that plants could regulate the rhizosphere microbiome composition and that different rhizosphere microbial composition has different effects on plant traits, such as growth and disease resistance (Hou et al., 2021). Few studies have also investigated the relationship between rhizosphere microbes and *C*. *sativus* or other medicinal plants. Some rhizosphere microbes, including *Pseudomonas aeruginosa*, *Brevibacterium frigoritolerans*, *Alcaligenes faecalis* subsp. *phenolicus*, and *Bacillus aryabhattai*, were found to have plant growth properties and effectively control the growth of pathogenic fungi of *C. sativus* (Hu et al., 2021; Rasool et al., 2021). In this study, the microbial structure related to rot disease resistance in the rhizosphere of *C*. *sativus* was preliminarily revealed by metagenomic sequencing.

Microbial diversity and composition were different in *C*. *sativus* from different habitats, but those in the same habitat tended to be the same. Rhizosphere microorganisms of *Pseudostellaria heterophylla* were analyzed using phospholipid fatty acid analysis, and great differences were found in the microbial composition and structure of *P*. *heterophylla* from different habitats (Zou et al., 2018). This is related to the weather and planting methods in different places, and it is also regulated by the host (Gu et al., 2022; Silva et al., 2022; Zhong et al., 2022). In this study, Hypocreales and Burkholderiales were enriched in the microbial communities in the groups with high disease resistance. Among these, the common biocontrol fungi *Talaromyces* and *Trichoderma* belong to Hypocreales (Sood et al., 2020; Thambugala et al., 2020). Also, Proteobacteria was enriched in the high resistance groups, which was similar to the findings of Trivedi et al. (2012), who analyzed the citrus rhizosphere and found that it was enriched in Proteobacteria when plants were healthy. In addition, the abundance of *F*. *oxysporum* in different samples was compared, and the groups with high disease resistance had higher *F*. *oxysporum* abundance, which might be because of the higher tolerance of the microbial communities in these groups to *F*. *oxysporum*. This is similar to an analysis of ginseng in which the abundance of *Fusarium* in healthy ginseng was higher, suggesting nutritional competition between *Fusarium* and another pathogen *Ilyonectria* (Liu et al., 2019). Pseudomonadales, Corynebacteriales, and Propionibacteriales had higher abundance in the group with high disease resistance. A previous study analyzed the rhizosphere soil of common bean with *F*. *oxysporum*-resistant microorganisms and found that it had a higher abundance of Pseudomonadaceae than the rhizosphere soil of susceptible varieties (Mendes et al., 2018). Corynebacteriales and Propionibacteriales, belonging to Actinobacteria, are common groups that produce antibiotics, cellulase, and other antibacterial substances (Palaniyandi et al., 2013). In addition, there was a high abundance of *Oidiodendron* in Rs_JD, suggesting that Rs_JD has high disease resistance because *Oidiodendron* was found to produce biocontrol substances including polyketides and antibiotics (Andersen et al., 1983; Navarri et al., 2017). However, studies of *Oidiodendron* and its biocontrol activity in plant disease are few, and it may be a highly underestimated biocontrol strain.

Correlation analysis between rhizosphere microbes and rot disease resistance revealed that *Asanoa* and *Moniliophthora* were the most likely genera to enhance host rot resistance. *Asanoa* is a rare bacterial genus within Micromonosporaceae. *Asanoa* was previously isolated as a rhizosphere microbe of the alpine medicinal plant *Leontopodium nivale* subsp. *alpinum*, but its activity was not investigated (Oberhofer et al., 2019). Using 16s rDNA sequencing technology, *Asanoa* was found as an endophyte of *L*. *nivale* subsp. *alpinum*, suggesting that it plays a role in resisting rot disease as a colonizing endophyte. However, this bacterial species was not isolated from *C. sativus*, and its biocontrol activity against corm rot disease of *C. sativus* needs to be further studied. In addition, no studies discuss the relationship between *Moniliophthora* and *C*. *sativus*, but because *Moniliophthora* is a pathogen of *Theobroma cacao* (Ali et al., 2021), its specific activity in *C*. *sativus* requires further experimental investigation. In the range of isolated microbes, the genera most closely related to disease resistance were *Enterobacter* (bacteria) and *Talaromyces* (fungi). *Enterobacter* can improve host disease resistance, promote plant growth, and enhance abiotic stress resistance (Ajmal et al., 2022; Mukherjee et al., 2022; Panebianco et al., 2022). *Talaromyces* is ubiquitously used as biocontrol agents worldwide (Thambugala et al., 2020). In addition, RDA showed that *Burkholderia* abundance had a high positive correlation with AK and a high negative correlation with RLA, indicating a correlation among disease resistance, soil physicochemical properties, and microbial composition. Lower pH and higher AK may be beneficial to the health of *C*. *sativus*. Both environment and rhizosphere microbiome may directly affect the health of *C*. *sativus* corms, or they may play a role by affecting each other (Ren et al., 2020; Tian et al., 2021b).

The possible mechanism of the resistance of rhizosphere microorganisms to plant disease is synthesizing pathogen-antagonizing compounds (Jiao et al., 2021). The production of antibiotics, polyketides, chitinases, etc. is a sign that microorganisms can help host disease resistance. *Bacillus thuringiensis* has become the main microorganism used in biological control because it produces chitinases (Melo et al., 2016). Rs_QC, a group with high disease resistance, had a higher abundance of chitinase-related genes, whereas the other high disease resistance groups Rs_QC and Rs_JD had a high abundance of β-1,3-glucanase-related genes, which might be the reason for their high disease resistance. In addition, Rs_XZ with high disease resistance had a high abundance of polyketide synthesis genes (**Figure S4**). k00128 and k00626 (acetyl CoA C-acetyltransferase, EC: 2.3.1.9) are pathways related to polyketide synthesis. There was a high abundance of k00128 in all high resistance groups, whereas Rs_XZ had a high k00626 pathway abundance, which was significantly higher than that in the other groups (**Figure S5**). In addition, several groups were compared in the KEGG pathways (level 2), and the gene abundance of cell motility was higher in the high resistance groups (**Figure S6**). *B. seminalis* in which the antagonistic effect against pathogens was reduced by mutation had low cell motility, indicating that cell motility plays an important role in microbial antagonism (Zhang et al., 2020).

Fungicides and host resistance cannot often offer adequate and sustainable control of soil-borne diseases (Weller et al., 2002). Therefore, four antagonists with antifungal activity against pathogenic *F. oxysporum* were isolated and screened. *T. yunnanense* SR38 and *Talaromyces* sp. SR55 belonging to Hypocreales, *B. gladioli* SR379 from Burkholderiales, and *Enterobacter* sp. SR343 belonging to Proteobacteria were microbial taxa enriched in all high resistance groups, and their related disease resistance activities have already been reported in other studies (Sharma et al., 2015; Luo et al., 2021; Ahmad et al., 2022; Zhao et al., 2022). A new *Talaromyces* strain DYM25 was isolated from the Yap Trench and identified as a biocontrol agent against *Fusarium* wilt of cucumber (Luo et al., 2021). However, *Enterobacter*, an occasional pathogen, has not been reported in *C*. *sativus* (Zhang et al., 2022). *Enterobacter cloacae* isolated from *C*. *sativus* could produce cellulase and indole acetic acid, which usually have the potential for disease resistance and growth promotion (Sharma et al., 2015). In addition, it has been found that *B. gladioli* E39CS3 isolated from *C*. *sativus* corms can significantly improve the disease resistance of *C*. *sativus*, and verified that *B. gladioli* can produce chitinase and β-1,3-glucanase (Ahmad et al., 2022). In addition, because of the complexity of practical application, single biocontrol antagonists are usually considered to have the disadvantages of inadequate colonization and inefficient inhibition (Niu et al., 2020). They may have the problems of poor colonization rate and stability, including poor survival rate in soil, poor compatibility with host, and influence of original host microorganisms (Martínez-Viveros et al., 2010; Sarma et al., 2015; Vejan et al., 2016). Multi-strain biological control agents may have unique advantages in dealing with these problems (Niu et al., 2020). Therefore, correlation network analysis was used to analyze the relationships among genera based on metagenomic data, and *Trichoderma* (at the center of the network) and *Paenibacillus* (at the edge of the network) were combined (*T. yunnanense* SR38 + *Paenibacillus peoriae* SR235), which showed a 1.5-fold biocontrol effect compared with SR379 (unpublished data). Similarly, *T. virens* Gl006 and *Bacillus velezensis* Bs006 in combination can control *Fusarium* wilt of Cape gooseberry better (Izquierdo-García et al., 2020). Therefore, studies on multi-strain antagonists, one of the development directions of biocontrol, should be increased in follow-up research. More investigation is needed to understand the interactions between rhizosphere microorganisms and *C*. *sativus* to improve *C*. *sativus* yields and increase its resilience to biotic and abiotic stresses. To more comprehensively reveal the effective mechanism of rhizosphere microorganisms, the relationship between rhizospheric and endophytic microorganisms will be considered in follow-up research.

## 5 Conclusion

Soil chemical properties and corm susceptibility influenced rhizosphere microbiome of *C. sativus*. pH was the closest environmental factor related to microbial communities, followed by Cr and AN. The order Burkholderiales was a vital taxon that kept *C*. *sativus* corms healthy. Other bacterial and fungal taxa were also differentially distributed in high and low disease resistance groups. Such differences played an important role in improving *C*. *sativus* corm rot disease resistance. Four rhizosphere isolates displayed biocontrol effects against *C*. *sativus* corm rot disease. These antagonists are promising isolates to be developed as biological agents to control corm rot disease of *C. sativus*. This study can serve as a reference for the exploration of the relationship between rhizosphere microorganisms and the host. However, further experiments are required to gain insight into the molecular mechanisms of these antagonists and explore the impact of microbial inoculation on biological activities of soil under natural conditions.

## Data availability statement

The original contributions presented in the study are publicly available. This data can be found here: NCBI, PRJNA888150.

## Author contributions

LQ and BZ designed the research. JZ, YZ, and QH performed the experiments and analyzed the data. JZ and JL wrote the manuscript. All authors critically revised the manuscript and approved the final version.

## Funding

This study was supported by the National Natural Science Foundation of China (82003896, 81673528), Natural Science Foundation of Zhejiang Province (LQ21H280003), Young Innovative Talents Project of Zhejiang Medical Health Science and Technology (2022RC052), and Talent Projects of Zhejiang Chinese Medical University (2021ZR09).

## Acknowledgments

We thank Peng Li and Shaowen Liu in Shanghai Majorbio Bio-pharm Technology Co., Ltd. for help in sequencing.

## Conflict of interest

The authors declare that the research was conducted in the absence of any commercial or financial relationships that could be construed as a potential conflict of interest.

## Publisher’s note

All claims expressed in this article are solely those of the authors and do not necessarily represent those of their affiliated organizations, or those of the publisher, the editors and the reviewers. Any product that may be evaluated in this article, or claim that may be made by its manufacturer, is not guaranteed or endorsed by the publisher.
