# Supplementary figures and images for: Rhizosphere microorganisms of Crocus sativus as antagonists against pathogenic Fusarium oxysporum
Source: Front Plant Sci. 2022 Nov 22;13:1045147. doi: 10.3389/fpls.2022.1045147 (PMC9722746; doi:10.3389/fpls.2022.1045147)

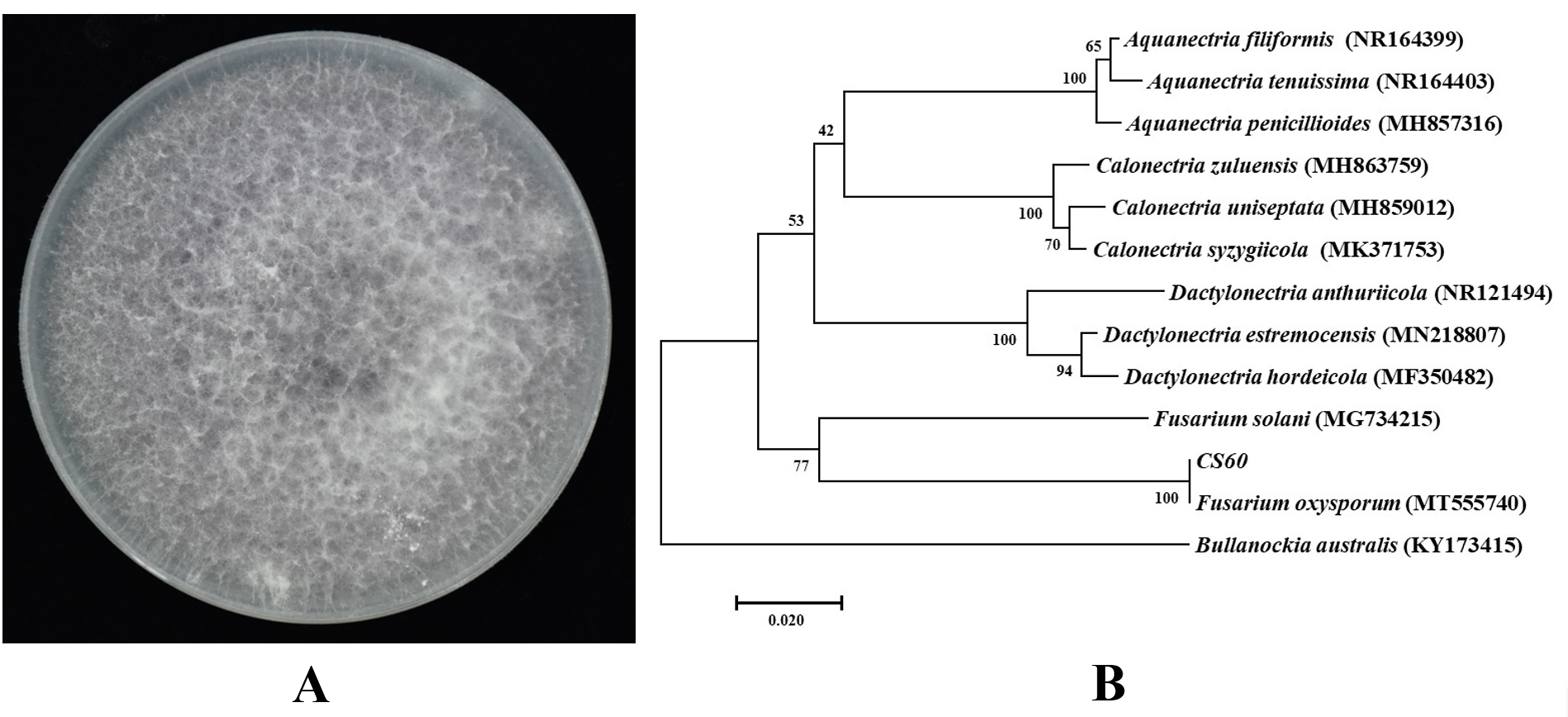

Supplement: Supplementary file 1 [file Image_1.jpeg]

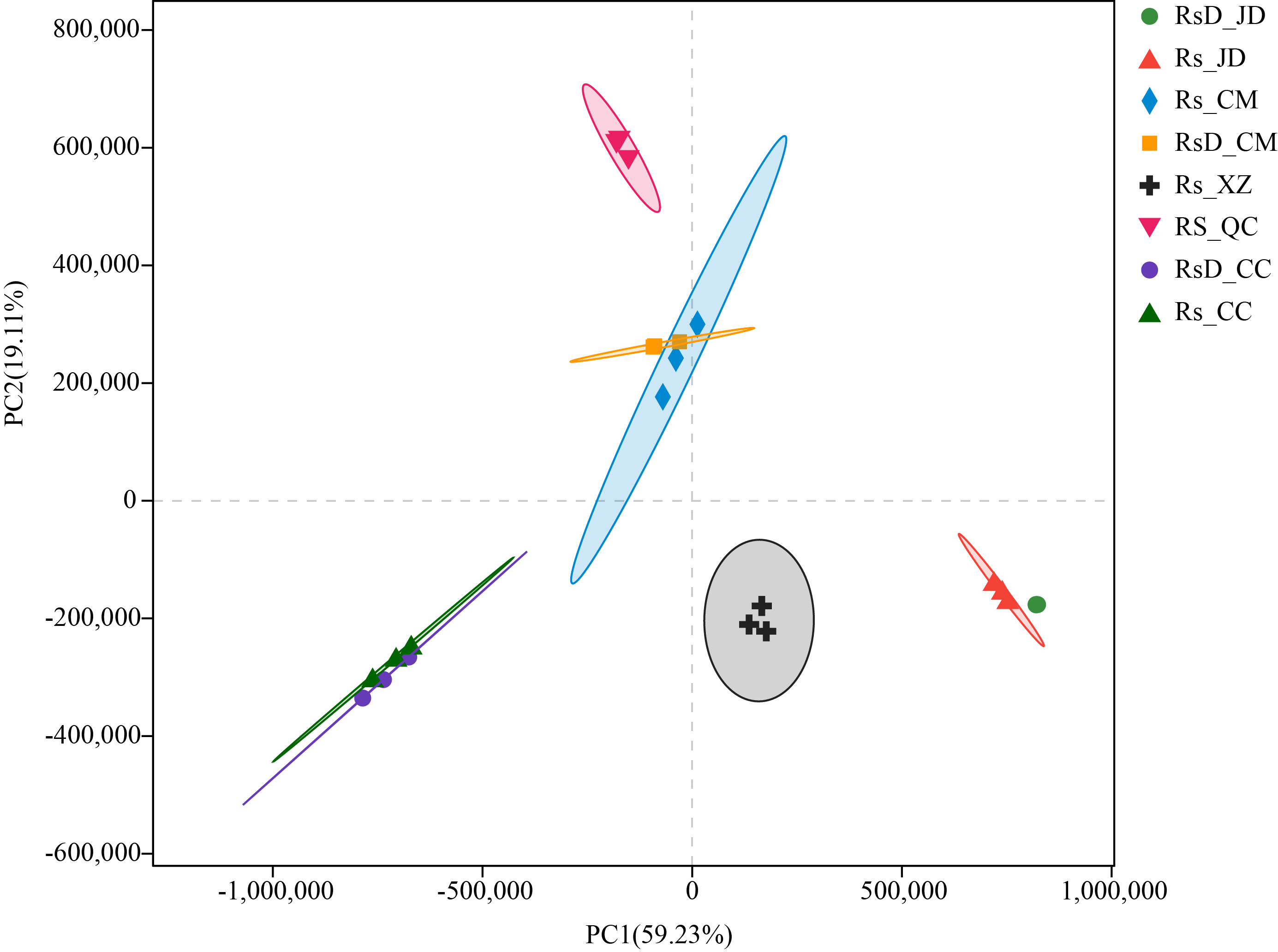

Supplement: Supplementary file 2 [file Image_2.tif]

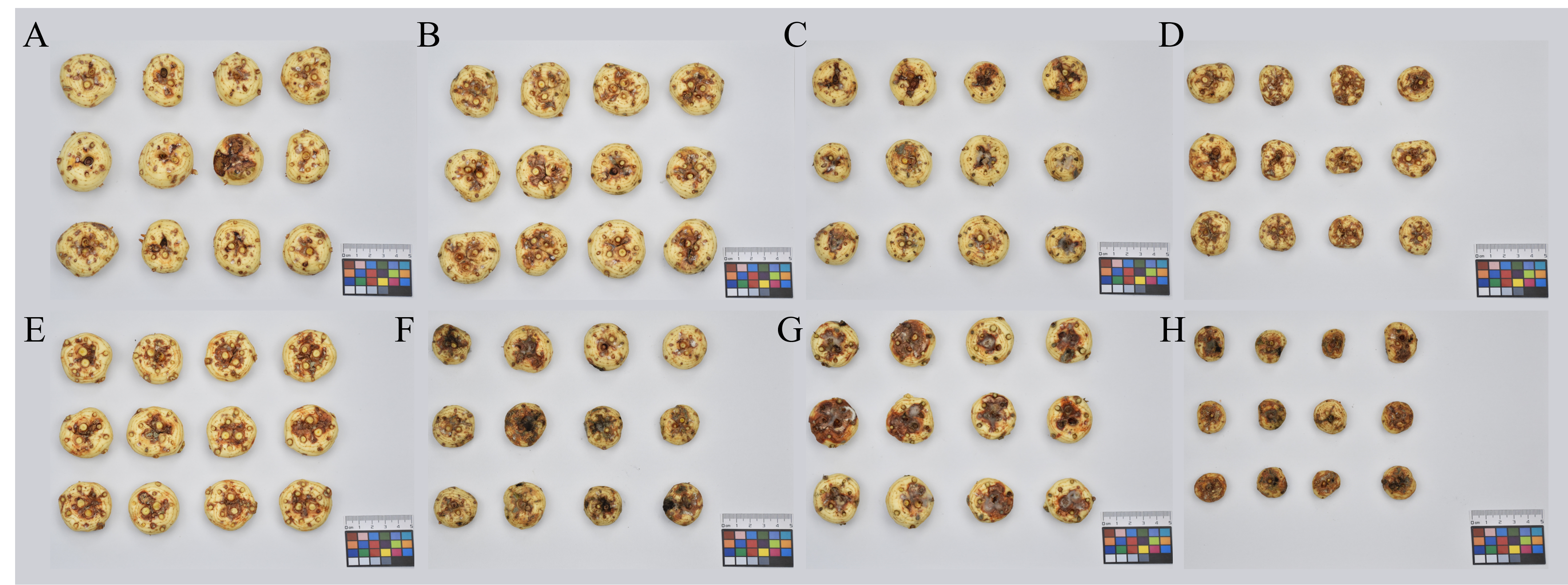

Supplement: Supplementary file 3 [file Image_3.png]

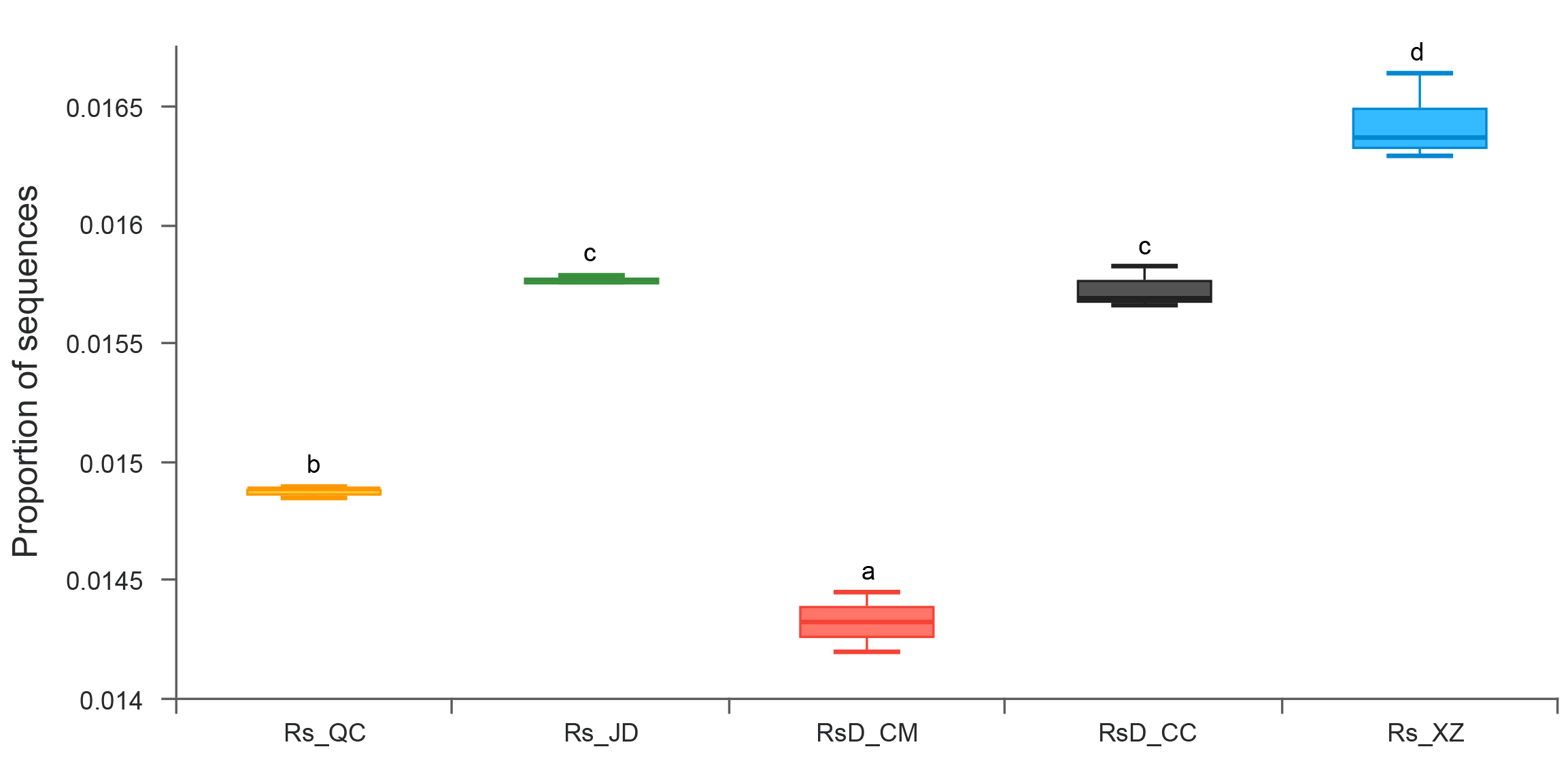

Supplement: Supplementary file 4 [file Image_4.tif]

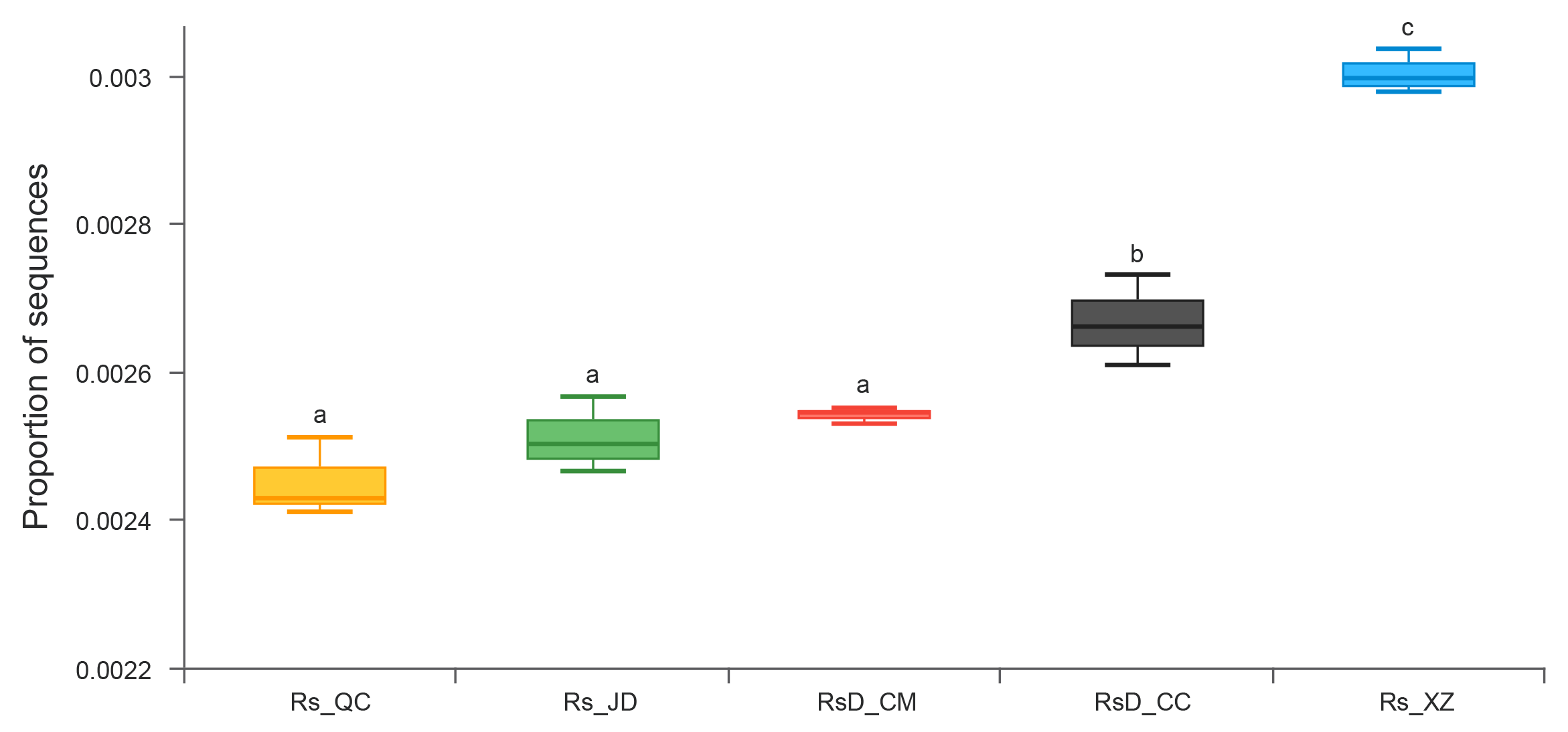

Supplement: Supplementary file 5 [file Image_5.tif]

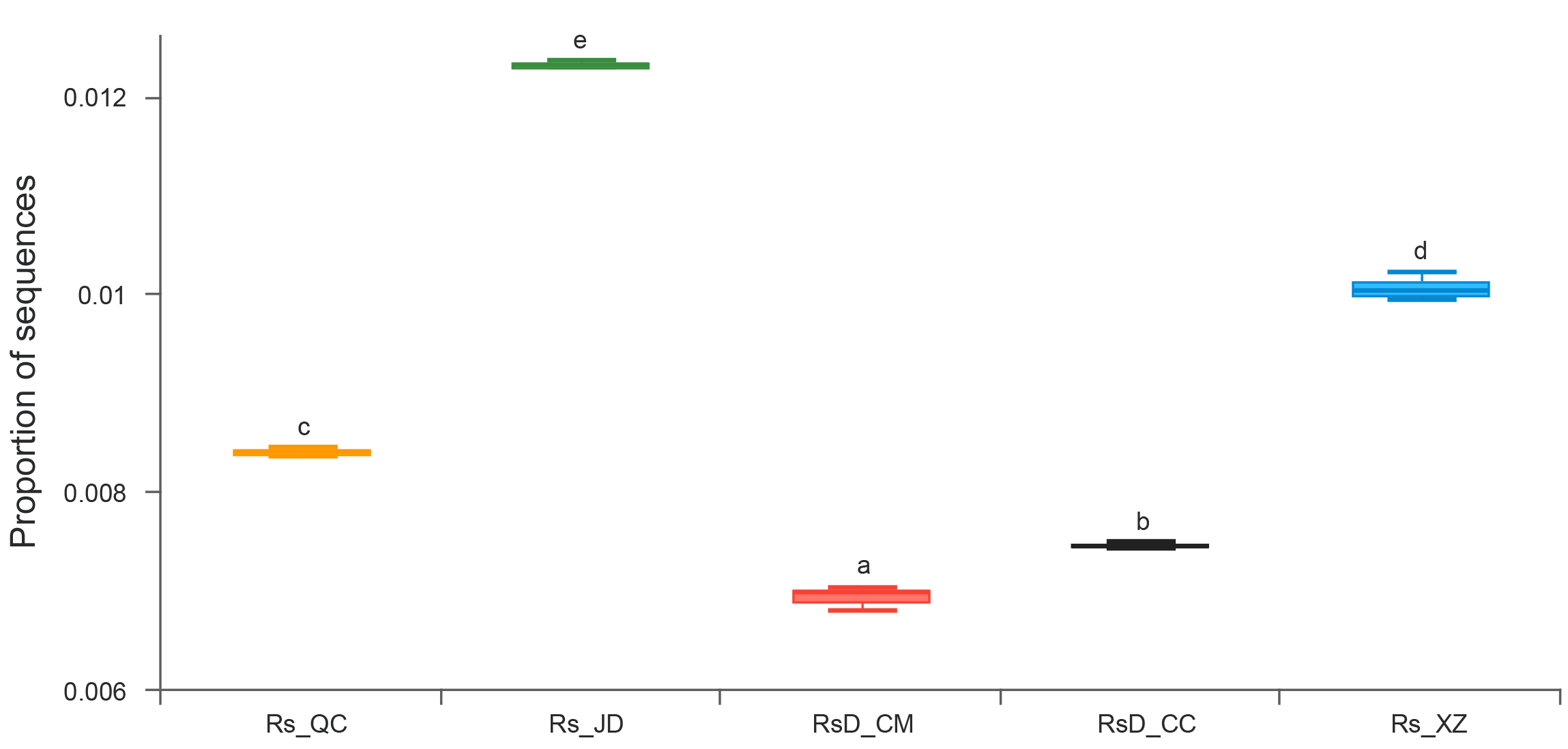

Supplement: Supplementary file 6 [file Image_6.tif]
