# Supplementary material for: Rhizosphere microorganisms of Crocus sativus as antagonists against pathogenic Fusarium oxysporum
Source: Front Plant Sci. 2022 Nov 22;13:1045147. doi: 10.3389/fpls.2022.1045147 (PMC9722746; doi:10.3389/fpls.2022.1045147)
Supplement: Supplementary file 7 [file DataSheet_1.docx]

Table S1 Metagenomic sequencing and data processing of *Crocus sativus* rhizospheric soils

| Samples | Raw reads information | | | | | Clean reads information | | | | Assembly results | | | | ORFs results | | | | |
| --- | --- | --- | --- | --- | --- | --- | --- | --- | --- | --- | --- | --- | --- | --- | --- | --- | --- | --- |
|  | Samples abbreviation | Insert Size (bp) | Read length (bp) | Raw reads | Raw base (bp) | Clean reads | Clean base(bp) | Percent in raw reads(%) | Percent in raw bases(%) | Contigs | Contigs bases(bp) | Max(bp) | Min(bp) | ORFs | Total Length(bp) | Average Length(bp) | Max(bp) | Min(bp) |
| *C. sativus* rhizospheric soil from Chengcheng 1 | Rs_CC1 | 525 | 150 | 89692026 | 13543495926 | 87009900 | 13118747205 | 97.00962714 | 96.86381771 | 913250 | 527571643 | 112183 | 300 | 1348867 | 562316919 | 416.88 | 10196 | 100 |
| *C. sativus* rhizospheric soil from Chengcheng 2 | Rs_CC2 | 525 | 150 | 87841670 | 13264092170 | 85664402 | 12920199113 | 97.52137226 | 97.40733815 | 885842 | 520840536 | 82802 | 300 | 1130557 | 486543923 | 430.36 | 11244 | 100 |
| *C. sativus* rhizospheric soil from Chengcheng 3 | Rs_CC3 | 525 | 150 | 99562456 | 15033930856 | 97273892 | 14671723169 | 97.70137852 | 97.59073199 | 1052471 | 614480699 | 137241 | 300 | 1102547 | 455395433 | 413.04 | 23430 | 100 |
| *C. sativus* rhizospheric soil from Chongming 1 | Rs_CM1 | 520 | 150 | 86121562 | 13004355862 | 83695406 | 12620088940 | 97.18287042 | 97.04509069 | 659558 | 392827850 | 51606 | 300 | 1392728 | 570270868 | 409.46 | 10715 | 100 |
| *C. sativus* rhizospheric soil from Chongming 2 | Rs_CM2 | 520 | 150 | 81296380 | 12275753380 | 78991542 | 11909873118 | 97.16489467 | 97.01948833 | 561501 | 313359310 | 25423 | 300 | 1107932 | 483386768 | 436.3 | 14600 | 100 |
| *C. sativus* rhizospheric soil from Chongming 3 | Rs_CM3 | 520 | 150 | 88262124 | 13327580724 | 86052808 | 12973021728 | 97.49686967 | 97.33965974 | 666267 | 386542962 | 104019 | 300 | 971292 | 432929456 | 445.73 | 22464 | 100 |
| *C. sativus* rhizospheric soil from Jiande 1 | Rs_JD1 | 546 | 150 | 93054554 | 14051237654 | 90043276 | 13576588846 | 96.76396493 | 96.62201423 | 845566 | 575529019 | 158476 | 300 | 1112224 | 492979438 | 443.24 | 19412 | 100 |
| *C. sativus* rhizospheric soil from Jiande 2 | Rs_JD2 | 525 | 150 | 86609450 | 13078026950 | 84008184 | 12666106899 | 96.99655638 | 96.85028902 | 767511 | 518008324 | 133737 | 300 | 1336835 | 565078372 | 422.7 | 12492 | 100 |
| *C. sativus* rhizospheric soil from Jiande 3 | Rs_JD3 | 525 | 150 | 93333808 | 14093405008 | 90923600 | 13700664456 | 97.41764742 | 97.21330259 | 884384 | 572843721 | 162287 | 300 | 1210458 | 495806834 | 409.6 | 7817 | 100 |
| *C. sativus* rhizospheric soil from Qiaocheng 1 | Rs_QC1 | 535 | 150 | 89692316 | 13543539716 | 87408670 | 13179409815 | 97.45391121 | 97.31141261 | 972636 | 604086203 | 233643 | 300 | 1000113 | 410392355 | 410.35 | 10432 | 100 |
| *C. sativus* rhizospheric soil from Qiaocheng 2 | Rs_QC2 | 535 | 150 | 97318388 | 14695076588 | 94876262 | 14311812044 | 97.49058112 | 97.39188468 | 1054843 | 666906687 | 233643 | 300 | 998778 | 438020144 | 438.56 | 11244 | 100 |
| *C. sativus* rhizospheric soil from Qiaocheng 3 | Rs_QC3 | 535 | 150 | 103060218 | 15562092918 | 100515734 | 15156614035 | 97.53107062 | 97.39444505 | 1267172 | 759801276 | 242950 | 300 | 1225818 | 513001643 | 418.5 | 12492 | 100 |
| *C. sativus* rhizospheric soil from Xiuzhou 1 | Rs_XZ1 | 535 | 150 | 104208360 | 15735462360 | 101865460 | 15360839862 | 97.75171589 | 97.61924696 | 1117723 | 689784057 | 146593 | 300 | 817692 | 338870087 | 414.42 | 16689 | 100 |
| *C. sativus* rhizospheric soil from Xiuzhou 2 | Rs_XZ2 | 535 | 150 | 91901384 | 13877108984 | 89798652 | 13540776085 | 97.71196917 | 97.5763475 | 958299 | 587158508 | 175868 | 300 | 1203527 | 508492168 | 422.5 | 30927 | 100 |
| *C. sativus* rhizospheric soil from Xiuzhou 3 | Rs_XZ3 | 535 | 150 | 88937452 | 13429555252 | 86724288 | 13076622369 | 97.51155003 | 97.37196894 | 927857 | 586607815 | 205200 | 300 | 1138077 | 465821851 | 409.31 | 10513 | 100 |
| Degenerative *C. sativus* rhizospheric soil from Chengcheng 1 | RsD_CC1 | 520 | 150 | 99840790 | 15075959290 | 97086438 | 14643129095 | 97.2412558 | 97.12900395 | 1123335 | 646991675 | 105651 | 300 | 1301199 | 532607776 | 409.32 | 14800 | 100 |
| Degenerative *C. sativus* rhizospheric soil from Chengcheng 2 | RsD_CC2 | 520 | 150 | 89821618 | 13563064318 | 87590878 | 13205796515 | 97.5164776 | 97.36587695 | 972545 | 558511446 | 137284 | 300 | 1039913 | 427360971 | 410.96 | 14986 | 100 |
| Degenerative *C. sativus* rhizospheric soil from Chengcheng 3 | RsD_CC3 | 520 | 150 | 98546770 | 14880562270 | 96124678 | 14499003819 | 97.54219037 | 97.43585999 | 1072091 | 633362583 | 105648 | 300 | 1557939 | 633561786 | 406.67 | 12492 | 100 |
| Degenerative *C. sativus* rhizospheric soil from Chongming 1 | RsD_CM1 | 520 | 150 | 82611666 | 12474361566 | 80445938 | 12124657057 | 97.37842353 | 97.19661397 | 616592 | 350582405 | 29842 | 300 | 690449 | 275817592 | 399.48 | 5784 | 100 |
| Degenerative *C. sativus* rhizospheric soil from Chongming 2 | RsD_CM2 | 547 | 150 | 103226472 | 15587197272 | 100342492 | 15136002566 | 97.20616239 | 97.10535064 | 796650 | 464628977 | 53606 | 300 | 1396621 | 589944893 | 422.41 | 30927 | 100 |
| Degenerative *C. sativus* rhizospheric soil from Chongming 3 | RsD_CM3 | 535 | 150 | 104735856 | 15815114256 | 101959996 | 15381274683 | 97.34965645 | 97.25680405 | 829425 | 484523977 | 64989 | 300 | 825032 | 336738237 | 408.15 | 7650 | 100 |
| Degenerative *C. sativus* rhizospheric soil from Jiande 1 | RsD_JD1 | 525 | 150 | 94053868 | 14202134068 | 91461086 | 13784624520 | 97.24330104 | 97.06023372 | 739732 | 523070591 | 220175 | 300 | 1189364 | 511572844 | 430.12 | 30601 | 100 |
| Degenerative *C. sativus* rhizospheric soil from Jiande 2 | RsD_JD2 | 525 | 150 | 97669600 | 14748109600 | 94868272 | 14301687505 | 97.13183222 | 96.97302158 | 855345 | 582336232 | 141802 | 300 | 765133 | 310340747 | 405.6 | 6393 | 100 |
| Degenerative *C. sativus* rhizospheric soil from Jiande 3 | RsD_JD3 | 525 | 150 | 98115158 | 14815388858 | 95203346 | 14358729825 | 97.03225061 | 96.91767096 | 827444 | 562934015 | 162287 | 300 | 1065491 | 462595260 | 434.16 | 13900 | 100 |

Table S2 Relative inhibition rate of antagonists against *F. oxysporum* growth on PDA plate

| Genus | Correlation | P-value | Strain | Accession numbers in Genbank | Relative inhibiting rate |
| --- | --- | --- | --- | --- | --- |
| g__*Enterobacter* | -0.649018674 | 0.000601065 | *Enterobacter cloacae* 202 | MZ026404 | 14.1578% |
|  |  |  | *Enterobacter* sp. 343 | MZ026442 | 34.2857% |
| g__*Talaromyces* | -0.639822053 | 0.000760272 | *Talaromyces* sp. 55 | OL307963 | 68.0817% |
|  |  |  | *Talaromyces* sp. 25 | OL307948 | 7.7012% |
|  |  |  | *Talaromyces* sp. 18 | OL307944 | 21.1614% |
|  |  |  | *Talaromyces* sp. 27 | OL307949 | 50.9663% |
|  |  |  | *Talaromyces minioluteus* 20 | OL307945 | 45.1976% |
| g__*Mucor* | -0.582648656 | 0.002811114 | *Mucor* sp. 103 | OL307976 | 53.1865% |
| g__*Burkholderia* | -0.556931354 | 0.004700709 | *Burkholderia gladioli* 379 | MZ026451 | 35.2381% |
| g__*Fusarium* | -0.528033972 | 0.007997812 | *Fusarium* sp. 01 | OL307935 | 50.0048% |
|  |  |  | *Fusarium* sp. 62 | OL307966 | 29.8144% |
|  |  |  | *Fusarium* sp. 09 | OL307939 | -2.8747% |
|  |  |  | *Fusarium* sp. 99 | OL307973 | 52.8891% |
|  |  |  | *Fusarium* sp. 89 | OL307970 | 53.8506% |
|  |  |  | *Fusarium* sp. 61 | OL307965 | 30.8437% |
|  |  |  | *Fusarium* sp. 11 | OL307940 | 40.4192% |
|  |  |  | *Fusarium* sp. 94 | OL307971 | 27.8915% |
|  |  |  | *Fusarium* sp. 97 | OL307972 | 10.5855% |
|  |  |  | *Fusarium* sp. 15 | OL307941 | -0.9518% |
| g__*Bacillus* | -0.50439066 | 0.011956939 | *Bacillus* sp. 115 | MZ026391 | 5.3081% |
|  |  |  | *Bacillus idriensis* 163 | MZ026397 | 13.2728% |
|  |  |  | *Bacillus drentensis* 245 | MZ026409 | -16.1905% |
|  |  |  | *Bacillus* sp. 253 | MZ026411 | 17.6977% |
|  |  |  | *Bacillus* sp. 281 | MZ026424 | 3.5381% |
|  |  |  | *Bacillus* sp. 296 | MZ026430 | -20.9524% |
|  |  |  | *Bacillus* sp. 375 | MZ026450 | 0.8832% |
|  |  |  | *Bacillus paramycoides* 380 | MZ026452 | 14.1578% |
|  |  |  | *Bacillus* sp. 289 | MZ026427 | -28.5714% |
| g__*Rhizobium* | -0.499136591 | 0.013026366 | *Rhizobium herbae* 365 | MZ026447 | -14.2857% |
| g__*Trichoderma* | -0.472866244 | 0.019617885 | *Trichoderma* sp. 02 | OL307936 | 90.4245% |
|  |  |  | *Trichoderma* *yunnanense* 38 | OL307957 | 91.8277% |
|  |  |  | *Trichoderma* sp. 88 | OL307969 | 83.6554% |

Table S3 Disease resistance test of *Crocus sativus* corm from various habitats (relative lesion area, RLA)

| Rs_QC | | | Rs_JD | | | RsD_JD | | | Rs_XZ | | | Rs_CM | | | RsD_CM | | | Rs_CC | | | RsD_CC | | |
| --- | --- | --- | --- | --- | --- | --- | --- | --- | --- | --- | --- | --- | --- | --- | --- | --- | --- | --- | --- | --- | --- | --- | --- |
| Rs_QC1 | Rs_QC2 | Rs_QC3 | Rs_JD1 | Rs_JD2 | Rs_JD3 | RsD_JD1 | RsD_JD2 | RsD_JD3 | Rs_XZ1 | Rs_XZ2 | Rs_XZ3 | Rs_CM1 | Rs_CM2 | Rs_CM3 | RsD_CM1 | RsD_CM2 | RsD_CM3 | Rs_CC1 | Rs_CC2 | Rs_CC3 | RsD_CC1 | RsD_CC2 | RsD_CC3 |
| 16.68% | 16.27% | 31.04% | 13.26% | 19.58% | 28.54% | 23.02% | 34.91% | 18.36% | 7.16% | 16.19% | 21.66% | 15.53% | 45.46% | 36.43% | 47.98% | 47.30% | 40.93% | 59.15% | 12.67% | 63.13% | 66.11% | 39.12% | 62.54% |
| 17.07% | 27.09% | 15.43% | 27.79% | 27.26% | 6.84% | 22.44% | 23.70% | 14.97% | 37.31% | 25.01% | 12.80% | 24.46% | 34.01% | 40.79% | 76.89% | 34.99% | 71.68% | 42.39% | 11.43% | 57.41% | 71.47% | 77.64% | 46.34% |
| 20.42% | 15.06% | 25.53% | 10.58% | 10.72% | 16.92% | 66.61% | 34.67% | 58.17% | 22.93% | 37.75% | 23.10% | 36.31% | 28.86% | 44.29% | 40.15% | 68.91% | 22.67% | 17.67% | 34.14% | 25.85% | 91.59% | 69.11% | 93.35% |
| 15.33% | 18.11% | 15.53% | 26.00% | 29.89% | 42.94% | 38.07% | 25.52% | 24.12% | 19.46% | 41.84% | 48.63% | 42.06% | 24.49% | 25.90% | 41.53% | 60.30% | 46.21% | 16.66% | 82.01% | 62.44% | 58.04% | 97.60% | 39.32% |
| 84.87% | 5.99% | 12.58% | 29.87% | 19.54% | 22.25% | 18.14% | 41.05% | 46.86% | 22.38% | 22.08% | 32.39% | 25.15% | 48.80% | 39.10% | 36.90% | 38.96% | 89.82% | 21.41% | 45.35% | 35.44% | 61.06% | 57.36% | 31.41% |
| 31.24% | 12.04% | 41.34% | 23.20% | 10.96% | 23.40% | 25.21% | 53.16% | 35.52% | 16.12% | 28.88% | 30.80% | 26.12% | 42.81% | 31.28% | 29.86% | 60.74% | 63.38% | 84.63% | 28.64% | 46.75% | 81.41% | 85.61% | 87.74% |
| 23.09% | 54.48% | 22.02% | 24.77% | 45.39% | 27.00% | 48.54% | 45.60% | 17.01% | 15.57% | 17.96% | 36.32% | 28.48% | 27.29% | 36.57% | 29.87% | 42.51% | 50.51% | 45.43% | 19.36% | 21.36% | 23.67% | 55.88% | 78.93% |
| 16.07% | 11.29% | 29.89% | 30.63% | 33.32% | 35.66% | 30.48% | 20.10% | 60.71% | 13.29% | 8.00% | 43.04% | 38.55% | 29.10% | 25.75% | 28.15% | 23.44% | 35.64% | 41.36% | 39.01% | 27.91% | 87.48% | 83.23% | 36.87% |
| 42.71% | 19.69% | 10.17% | 18.93% | 25.17% | 24.62% | 33.13% | 27.45% | 38.31% | 29.34% | 25.38% | 32.60% | 34.03% | 35.97% | 37.88% | 53.15% | 54.37% | 35.39% | 25.81% | 38.10% | 37.96% | 91.59% | 74.31% | 75.70% |
| 16.74% | 14.68% | 28.61% | 17.23% | 36.31% | 38.37% | 27.79% | 20.51% | 20.11% | 16.35% | 18.53% | 22.96% | 26.05% | 36.05% | 30.62% | 45.39% | 42.39% | 29.28% | 47.43% | 32.03% | 25.62% | 73.11% | 43.86% | 89.62% |
| 11.81% | 12.24% | 19.56% | 38.55% | 27.74% | 13.08% | 56.82% | 51.00% | 22.62% | 29.01% | 16.31% | 28.20% | 35.20% | 25.48% | 44.54% | 43.54% | 50.74% | 62.13% | 47.96% | 40.75% | 20.55% | 85.99% | 35.74% | 39.63% |
| 18.55% | 14.78% | 14.36% | 37.08% | 20.29% | 34.14% | 30.69% | 90.68% | 37.79% | 16.53% | 22.69% | 54.05% | 29.55% | 43.78% | 58.89% | 76.14% | 67.35% | 44.93% | 68.35% | 55.01% | 65.99% | 67.34% | 99.34% | 58.91% |
| 26.21% | 18.48% | 22.17% | 24.82% | 25.51% | 26.15% | 35.08% | 39.03% | 32.88% | 20.45% | 23.39% | 32.21% | 30.13% | 35.18% | 37.67% | 45.80% | 49.33% | 49.38% | 43.19% | 36.54% | 40.87% | 71.57% | 68.23% | 61.70% |
| 22.29 ± 3.87% | | | 25.50 ± 0.66% | | | 35.66 ± 3.12% | | | 25.35 ± 6.12% | | | 34.32 ± 3.84% | | | 48.17 ± 2.06% | | | 40.20 ± 3.37% | | | 67.17 ± 5.02% | | |

Table S4 Chemical properties of *Crocus sativus* rhizospheric soils

| Samples | pH | Available N (mg/kg) | Available P (mg/kg) | Available K (mg/kg) | Organic matter (g/kg) | Cr (mg/kg) |
| --- | --- | --- | --- | --- | --- | --- |
| Rs_JD | 6.2200±0.1476 | 245.4309±12.5519 | 12.2151±0.8619 | 611.9211±8.0631 | 21.4889±0.6290 | 60.0379±2.8320 |
| RsD_JD | 5.2033±0.1327 | 200.9487±5.9703 | 26.0723±0.4156 | 878.2902±15.5586 | 19.1336±1.0917 | 58.9072±2.0272 |
| Rs_XZ | 5.7267±0.0368 | 398.2807±8.7488 | 229.0374±6.1109 | 432.1608±9.0580 | 22.7311±0.8357 | 88.4237±1.0692 |
| Rs_QC | 7.9900±0.0616 | 147.7697±9.7840 | 97.0114±1.9820 | 658.5698±17.3262 | 21.7159±0.0718 | 54.1437±1.6640 |
| Rs_CC | 8.3300±0.0648 | 127.0171±8.3511 | 55.3904±1.2754 | 244.1246±10.5959 | 11.2515±0.08329 | 54.6112±1.6068 |
| RsD_CC | 8.3167±0.0340 | 77.0114±7.9984 | 51.2196±1.2958 | 231.8276±10.9521 | 11.3709±0.0782 | 52.119±1.9696 |
| Rs_CM | 8.0467±0.1212 | 113.6626±5.9923 | 33.0867±1.0182 | 83.1828±7.6858 | 24.1002±0.2092 | 49.7102±0.4635 |
| RsD_CM | 7.6167±0.0579 | 156.3628±10.0175 | 25.6253±0.8502 | 76.4477±5.2269 | 22.1719±0.0871 | 47.2093±1.3911 |

Table S5 Correlation coefficient of soil chemical properties with microbial communities in *Crocus sativus* rhizosphere

|  | RDA1 | RDA2 | r2 | p_values |
| --- | --- | --- | --- | --- |
| pH | -0.757248244 | 0.653127168 | 0.940011895 | 0.001 |
| Available N | 0.479924658 | -0.877309708 | 0.616621208 | 0.001 |
| Available P | -0.440811176 | -0.897599859 | 0.364436597 | 0.005 |
| Available K | 0.872859862 | -0.487970964 | 0.51573483 | 0.002 |
| Organic matter | 0.940375816 | 0.340137215 | 0.479898348 | 0.003 |
| Cr | 0.169704633 | -0.985494971 | 0.66602138 | 0.001 |
| area | -0.996154386 | 0.087615286 | 0.324406148 | 0.021 |

Table S6 Spearman correlation analysis between rhizosphere microbial abundance and *Crocus sativus* disease resistance (genus level, the top 100 shown)

| Genus name | Correlation | P-value |
| --- | --- | --- |
| g__*Bacteriovorax* | 0.890564759 | 5.56E-09 |
| g__*Lewinella* | 0.888130817 | 6.99E-09 |
| g__*Mariniradius* | 0.887203006 | 7.63E-09 |
| g__*Terrimonas* | 0.866921447 | 4.26E-08 |
| g__*Elusimicrobium* | 0.86486308 | 5.00E-08 |
| g__*Shimia* | 0.859040343 | 7.72E-08 |
| g__*Microscilla* | 0.838206304 | 3.18E-07 |
| g__*Rhodovulum* | 0.838024065 | 3.21E-07 |
| g__*Opitutus* | 0.824888892 | 7.10E-07 |
| g__*Colwellia* | 0.822440668 | 8.17E-07 |
| g__unclassified_f__Coxiellaceae | 0.813598016 | 1.33E-06 |
| g__*Intestinibacter* | 0.811632695 | 1.48E-06 |
| g__*Shewanella* | 0.806499649 | 1.94E-06 |
| g__*Perlucidibaca* | 0.805711767 | 2.02E-06 |
| g__*Desulfurella* | 0.803432869 | 2.28E-06 |
| g__*Asanoa* | -0.793364476 | 3.75E-06 |
| g__*Ottowia* | 0.781712776 | 6.46E-06 |
| g__*Sandaracinus* | 0.780229302 | 6.91E-06 |
| g__*Rhodoferax* | 0.77908517 | 7.27E-06 |
| g__unclassified_o__Verrucomicrobiales | 0.774975233 | 8.73E-06 |
| g__unclassified_p__Verrucomicrobia | 0.772348198 | 9.78E-06 |
| g__*Pilimelia* | 0.772316527 | 9.80E-06 |
| g__*Hydrocarboniphaga* | 0.761840059 | 1.52E-05 |
| g__*Bdellovibrio* | 0.759213025 | 1.70E-05 |
| g__*Aquamicrobium* | 0.755601066 | 1.96E-05 |
| g__*Mesoflavibacter* | 0.754944308 | 2.01E-05 |
| g__unclassified_f__Verrucomicrobiaceae | 0.748704886 | 2.57E-05 |
| g__*Curvibacter* | 0.746077851 | 2.84E-05 |
| g__*Marinimicrobium* | 0.746077851 | 2.84E-05 |
| g__*Terrimicrobium* | 0.746077851 | 2.84E-05 |
| g__*Wenzhouxiangella* | 0.738517912 | 3.78E-05 |
| g__*Blastopirellula* | 0.738196747 | 3.82E-05 |
| g__unclassified_f__Spongiibacteraceae | 0.734415868 | 4.39E-05 |
| g__*Pelagibacterium* | 0.732942678 | 4.63E-05 |
| g__unclassified_f__Rhodanobacteraceae | -0.727959335 | 5.53E-05 |
| g__*Massilia* | -0.727688608 | 5.58E-05 |
| g__*Lachnoanaerobaculum* | 0.717838036 | 7.84E-05 |
| g__*Roseibium* | 0.71718047 | 8.02E-05 |
| g__unclassified_f__Bacteroidaceae | 0.712162749 | 9.48E-05 |
| g__*Devriesea* | 0.709607958 | 0.000103053 |
| g__*Prosthecobacter* | 0.709529985 | 0.000103316 |
| g__*Moniliophthora* | -0.704658309 | 0.000120937 |
| g__*Gemmobacter* | 0.704045296 | 0.000123331 |
| g__*Octadecabacter* | 0.704045296 | 0.000123331 |
| g__*Hylemonella* | 0.704045296 | 0.000123331 |
| g__*Aliiroseovarius* | 0.702181918 | 0.000130862 |
| g__unclassified_o__Cytophagales | 0.700256991 | 0.000139062 |
| g__*Fluoribacter* | 0.697074013 | 0.000153608 |
| g__*Janthinobacterium* | -0.696164192 | 0.000158002 |
| g__*Lentisphaera* | 0.693838892 | 0.000169736 |
| g__*Congregibacter* | 0.693537157 | 0.000171313 |
| g__unclassified_p__Bacteroidetes | -0.693537157 | 0.000171313 |
| g__*Agaricus* | -0.692675532 | 0.000175887 |
| g__*Fibrobacter* | 0.692265884 | 0.000178098 |
| g__*Marinagarivorans* | 0.690910123 | 0.000185592 |
| g__*Holdemania* | 0.690648393 | 0.00018707 |
| g__*Puniceibacterium* | 0.688283088 | 0.000200897 |
| g__*Trueperella* | 0.686253055 | 0.000213467 |
| g__*Pseudophaeobacter* | 0.685954359 | 0.000215373 |
| g__*Mumia* | 0.685656053 | 0.000217292 |
| g__*Reinekea* | 0.684938394 | 0.000221968 |
| g__unclassified_f__Verrucomicrobia_subdivision_6 | 0.684491355 | 0.000224925 |
| g__*Raphidiopsis* | 0.683949419 | 0.000228556 |
| g__*Gilvimarinus* | 0.683029019 | 0.000234839 |
| g__*Acetobacterium* | 0.682160534 | 0.000240906 |
| g__*Turicibacter* | 0.682012093 | 0.000241956 |
| g__*Thiorhodovibrio* | 0.680401984 | 0.000253608 |
| g__*Neptuniibacter* | 0.679383915 | 0.000261225 |
| g__*Rubinisphaera* | 0.679236143 | 0.000262347 |
| g__*Tateyamaria* | 0.67792234 | 0.000272507 |
| g__*Lunatimonas* | 0.676608537 | 0.000283009 |
| g__*Caenimonas* | 0.675147915 | 0.000295095 |
| g__*Teredinibacter* | 0.673980931 | 0.000305071 |
| g__*Emticicia* | 0.67252088 | 0.000317964 |
| g__*Bavariicoccus* | -0.67229853 | 0.000319968 |
| g__*Pseudorhodobacter* | 0.670039522 | 0.000340963 |
| g__*Marinovum* | 0.667557116 | 0.000365404 |
| g__*Tatlockia* | 0.665218478 | 0.000389805 |
| g__unclassified_f__Oxalobacteraceae | -0.66478431 | 0.000394487 |
| g__*Pyxidicoccus* | 0.663909392 | 0.000404071 |
| g__unclassified_f__Parachlamydiaceae | 0.662589156 | 0.000418915 |
| g__*Simonsiella* | 0.662220687 | 0.000423141 |
| g__*Paraglaciecola* | 0.662012741 | 0.000425542 |
| g__*Flexithrix* | 0.656758672 | 0.000490263 |
| g__*Rhodobacter* | 0.656758672 | 0.000490263 |
| g__*Persicobacter* | 0.654986525 | 0.000513933 |
| g__*Allochromatium* | 0.65470119 | 0.000517835 |
| g__*Alcanivorax* | 0.654131637 | 0.000525701 |
| g__unclassified_f__Methylophilaceae | 0.65178805 | 0.000559164 |
| g__*Pectobacterium* | -0.65164628 | 0.000561246 |
| g__*Intrasporangium* | -0.651504602 | 0.000563333 |
| g__*Enterobacter* | -0.649018674 | 0.000601065 |
| g__*Ramlibacter* | 0.648877568 | 0.000603271 |
| g__*Phlebia* | -0.648834754 | 0.000603942 |
| g__*Marvinbryantia* | 0.647008045 | 0.000633161 |
| g__*Mastigocladus* | 0.646391068 | 0.000643302 |
| g__*Campylobacter* | 0.64155458 | 0.000727771 |
| g__*Rudanella* | 0.640996464 | 0.000738107 |
| g__unclassified_f__Succinivibrionaceae | 0.640790576 | 0.000741952 |

Table S7 Information of the data in CAZy

| Family | Family_Description | Class | Class_Description | Rs_XZ | RsD_CM | RsD_CC | Rs_JD | Rs_QC |
| --- | --- | --- | --- | --- | --- | --- | --- | --- |
| GH19 | chitinase (EC 3.2.1.14); lysozyme (EC 3.2.1.17) | GH | Glycoside Hydrolases | 0.1995±0.01408 | 0.2425±0.005557 | 0.1533±0.02078 | 0.2471±0.009452 | 0.3746±0.00315 |
| GH128 | beta-1,3-glucanase (EC 3.2.1.39) | GH | Glycoside Hydrolases | 0.07573±0.001077 | 0.09221±0.01096 | 0.1275±0.009674 | 0.1681±0.003376 | 0.1809±0.007116 |

Table S8 Information of the data in KEGG

| KO | KEGG Name | KO Description | Pathway ID | Pathway Description | Enzyme ID | Enzyme Description | Module ID | Module Description | Hyperlink | Rs_XZ | RsD_CM | RsD_CC | Rs_JD | Rs_QC |
| --- | --- | --- | --- | --- | --- | --- | --- | --- | --- | --- | --- | --- | --- | --- |
| K00128 | ALDH | aldehyde dehydrogenase (NAD+) [EC:1.2.1.3] | ko00010, ko00053, ko00071, ko00280, ko00310, ko00330, ko00340, ko00380, ko00410, ko00561, ko00620, ko00625, ko00903, ko00981 | Metabolism,Carbohydrate metabolism,Glycolysis / Gluconeogenesis; Metabolism,Carbohydrate metabolism,Ascorbate and aldarate metabolism; Metabolism,Lipid metabolism,Fatty acid degradation; Metabolism,Amino acid metabolism,Valine, leucine and isoleucine degradation; Metabolism,Amino acid metabolism,Lysine degradation; Metabolism,Amino acid metabolism,Arginine and proline metabolism; Metabolism,Amino acid metabolism,Histidine metabolism; Metabolism,Amino acid metabolism,Tryptophan metabolism; Metabolism,Metabolism of other amino acids,beta-Alanine metabolism; Metabolism,Lipid metabolism,Glycerolipid metabolism; Metabolism,Carbohydrate metabolism,Pyruvate metabolism; Metabolism,Xenobiotics biodegradation and metabolism,Chloroalkane and chloroalkene degradation; Metabolism,Metabolism of terpenoids and polyketides,Limonene and pinene degradation; Metabolism,Metabolism of terpenoids and polyketides,Insect hormone biosynthesis | 1.2.1.3 | Oxidoreductases,Acting on the aldehyde or oxo group of donors,With NAD+ or NADP+ as acceptor,aldehyde dehydrogenase (NAD+) | M00135 | Pathway module,Nucleotide and amino acid metabolism,Polyamine biosynthesis,GABA biosynthesis, eukaryotes, putrescine => GABA | http://www.genome.jp/dbget-bin/www_bget?ko:K00128 | 0.164±0.001952 | 0.1431±0.004301 | 0.1407±0.003001 | 0.1969±0.005582 | 0.1642±0.003113 |
| K02055 | ABC.SP.S | putative spermidine/putrescine transport system substrate-binding protein | ko02024 | Cellular Processes,Cellular community - prokaryotes,Quorum sensing | - | - | M00193 | Structural complex,Environmental information processing,Mineral and organic ion transport system,Putative spermidine/putrescine transport system | http://www.genome.jp/dbget-bin/www_bget?ko:K02055 | 0.1064±0.003045 | 0.08699±0.005454 | 0.0826±0.002092 | 0.1168±0.004168 | 0.0997±0.001482 |

Table S9 Relative lesion area of *Crocus sativus* corm treated with different antagonists

| Corm number | SR38 (1) | SR38 (2) | SR38 (3) | SR55 (1) | SR55 (2) | SR55 (3) | SR343 (1) | SR343 (2) | SR343 (3) | SR379 (1) | SR379 (2) | SR379 (3) | *F. oxysporum* (1) | *F. oxysporum* (2) | *F. oxysporum* (3) | carbendazim (1) | carbendazim (2) | carbendazim (3) |
| --- | --- | --- | --- | --- | --- | --- | --- | --- | --- | --- | --- | --- | --- | --- | --- | --- | --- | --- |
| 1 | 50.56% | 23.26% | 39.45% | 43.84% | 47.05% | 31.41% | 42.69% | 24.55% | 48.20% | 31.07% | 29.19% | 18.79% | 59.08% | 66.19% | 58.60% | 44.08% | 48.13% | 48.69% |
| 2 | 34.12% | 45.50% | 40.80% | 24.78% | 38.80% | 26.85% | 37.34% | 30.03% | 42.02% | 25.37% | 15.71% | 16.58% | 64.15% | 46.94% | 63.22% | 37.27% | 47.71% | 48.79% |
| 3 | 54.79% | 27.49% | 37.43% | 29.40% | 26.33% | 48.57% | 13.28% | 63.39% | 52.11% | 26.56% | 25.87% | 26.81% | 44.34% | 39.55% | 68.60% | 42.60% | 25.33% | 27.16% |
| 4 | 43.88% | 28.77% | 30.75% | 40.64% | 33.15% | 51.84% | 30.14% | 40.44% | 37.18% | 31.06% | 17.01% | 33.21% | 51.91% | 39.98% | 71.97% | 33.63% | 35.52% | 36.40% |
| 5 | 42.08% | 45.87% | 38.85% | 40.01% | 37.64% | 34.41% | 50.06% | 24.98% | 47.41% | 30.54% | 56.98% | 34.98% | 45.25% | 52.65% | 70.92% | 46.59% | 66.98% | 35.65% |
| 6 | 39.66% | 19.67% | 47.84% | 65.10% | 42.13% | 25.12% | 45.43% | 56.65% | 47.31% | 34.00% | 15.77% | 23.64% | 51.70% | 69.81% | 37.60% | 52.57% | 63.80% | 48.79% |
| 7 | 43.44% | 49.25% | 43.88% | 33.79% | 36.96% | 25.89% | 35.79% | 45.83% | 25.17% | 36.07% | 19.75% | 54.72% | 68.67% | 38.18% | 48.43% | 26.41% | 52.93% | 39.81% |
| 8 | 57.53% | 37.58% | 50.50% | 33.08% | 47.56% | 13.49% | 60.22% | 65.88% | 44.51% | 23.88% | 32.95% | 18.39% | 67.15% | 87.28% | 42.80% | 36.85% | 52.35% | 35.45% |
| 9 | 37.74% | 45.97% | 40.96% | 30.11% | 63.05% | 29.19% | 8.53% | 43.15% | 47.16% | 31.56% | 16.68% | 31.39% | 31.35% | 47.67% | 33.90% | 57.87% | 42.56% | 55.83% |
| 10 | 48.70% | 42.20% | 54.49% | 31.65% | 12.26% | 37.70% | 41.82% | 33.73% | 45.06% | 46.51% | 29.35% | 24.31% | 57.99% | 61.67% | 68.15% | 52.17% | 36.44% | 25.44% |
| 11 | 52.54% | 25.52% | 47.06% | 29.66% | 28.85% | 46.21% | 28.05% | 51.77% | 34.03% | 48.35% | 52.22% | 49.00% | 62.43% | 40.03% | 48.93% | 67.75% | 42.46% | 46.30% |
| 12 | 39.25% | 51.03% | 58.60% | 33.57% | 29.92% | 33.45% | 48.48% | 38.54% | 32.38% | 14.09% | 43.32% | 35.75% | 66.80% | 44.20% | 49.98% | 54.78% | 31.11% | 59.26% |
| 13 | 25.12% | 31.32% | 42.63% | 51.10% | 31.68% | 28.45% | 38.70% | 51.65% | 63.72% | 27.06% | 24.07% | 12.61% | 24.84% | 40.40% | 34.51% | 33.28% | 43.83% | 31.96% |
| 14 | 36.02% | 38.08% | 51.91% | 27.93% | 17.80% | 59.98% | 43.46% | 72.33% | 44.91% | 22.84% | 36.59% | 27.33% | 58.69% | 51.94% | 53.02% | 28.65% | 34.32% | 28.53% |
| 15 | 45.11% | 40.81% | 61.12% | 17.90% | 18.37% | 30.81% | 60.09% | 51.23% | 46.65% | 18.71% | 33.27% | 48.04% | 45.75% | 53.05% | 61.14% | 66.98% | 42.10% | 65.62% |
| 16 | 51.58% | 15.25% | 17.56% | 22.93% | 17.19% | 26.39% | 48.49% | 54.70% | 38.94% | 28.93% | 32.11% | 31.67% | 64.80% | 57.36% | 50.15% | 57.86% | 27.86% | 54.82% |
