# Supplementary material for: Rhizosphere microorganisms of Crocus sativus as antagonists against pathogenic Fusarium oxysporum
Source: Front Plant Sci. 2022 Nov 22;13:1045147. doi: 10.3389/fpls.2022.1045147 (PMC9722746; doi:10.3389/fpls.2022.1045147)
Supplement: Supplementary file 8 [file DataSheet_2.docx]

**Supplementary Figure Legends**

**Figure S1** The colony morphology of *Fusarium oxysporum* CS60 on PDA plate and phylogenetic position of *F. oxysporum* CS60 based on the ITS rRNA gene sequence

**Figure S2** Principal coordinate analysis (PCoA) of Euclidean distance of the communities in *Crocus* *sativus* samples.

**Figure S3** Symptoms of different *C*. *sativus* corm inoculated with *F*. *oxysporum*: (A) Rs_QC; (B) Rs_JD; (C) Rs_JX; (D) RsD_JD; (E) Rs_CM; (F) Rs_CC; (G) RsD_CM; (H) RsD_CC.

**Figure S4** The proportion of sequences related to metabolism of terpenoids and polyketides (Pathway Level2 level)

**Figure S5** The proportion of sequences related to K00626 (KEGG Orthology level)

**Figure S6** The proportion of sequences related to cell motility (Pathway Level2 level)
